# Revision of the *Gonioctena nivosa* species-group (Coleoptera, Chrysomelidae, Chrysomelinae) in the Holarctic region, with descriptions of two new species

**DOI:** 10.3897/zookeys.596.8725

**Published:** 2016-06-08

**Authors:** Hee-Wook Cho, Horst Kippenberg, Lech Borowiec

**Affiliations:** 1 Department of Biodiversity and Evolutionary Taxonomy, University of Wrocław, Przybyszewskiego 63/77, 51-148 Wrocław, Poland; 2 Langer Platz 21, D - 91074 Herzogenaurach, Germany

**Keywords:** Leaf beetles, taxonomic revision, geographic variation, ovoviviparity

## Abstract

The *Gonioctena nivosa* species-group of the genus *Gonioctena* Chevrolat, 1836 is defined and reviewed. It contains six species including two new to science: *Gonioctena gracilicornis* (Kraatz, 1879), *Gonioctena nivosa* (Suffrian, 1851), *Gonioctena norvegica* (Strand, 1936), *Gonioctena springlovae* (Bechyně, 1948), *Gonioctena amurensis* Cho & Borowiec, **sp. n.** and *Gonioctena jani* Cho & Borowiec, **sp. n.** Six new synonyms are proposed: *Gonioctena nivosa* (= *Gonioctena arctica alberta* Brown, 1952, **syn. n.**, *Phytodecta linnaeana bergrothi* Jacobson, 1901, **syn. n.**, Phytodecta linnaeanus var. mutatus Achard, 1924, **syn. n.**, Phytodecta linnaeanus var. simplex Achard, 1924, **syn. n.** and Phytodecta nivosa var. cedehensis Ronchetti, 1922, **syn. n.**) and *Gonioctena norvegica* (= *Gonioctena janovskii* Medvedev, 1976, **syn. n.**). Phytodecta flavicornis var. limbatipennis Achard, 1924 and Phytodecta nivosa var. bicolor Heyden, 1883 are removed from synonymy with *Gonioctena nivosa* (Suffrian, 1851) and are synonymized with *Gonioctena flavicornis* (Suffrian, 1851). Distribution maps, a key to species, color variation, geographic variation of male genitalia and host plants are provided. Ovoviviparity is newly recorded in *Gonioctena gracilicornis* and *Gonioctena nivosa*. Lectotypes are designated for *Gonioctena affinis*, *Gonioctena arctica*, *Gonioctena linnaeana bergrothi* and *Gonioctena nivosa*.

## Introduction

The genus *Gonioctena* Chevrolat, 1836 with about 100 described species in nine subgenera is one of the largest genera within the subfamily Chrysomelinae (Cho and Borowiec 2016). The nominotypical subgenus is the largest and contains 47 species that are widely distributed in the Holarctic and Oriental regions (Cho 2016). Many species of the nominotypical subgenus have received much attention due to their extremely high variability in coloration. Although the color pattern of several species has been revealed (Bechyně 1948, Silfverberg 1994b, V. L. Medvedev 2003, etc.), a similar color pattern between closely related sympatric species has produced a number of synonyms and misidentifications. The structure of male genitalia is generally used as the only source of reliable diagnostic characters. However, the shape of aedeagus is often geographically variable in several species with wide distributions or it is very similar between closely related species. The taxonomic status of these forms is still unclear. Kippenberg (2010) mentioned that 13 species of the subgenus *Gonioctena* s. str. in the catalogue of Palaearctic Coleoptera are characterized by the external morphology because they often have the similar shape of aedeagus. For example, the taxonomic status of the following taxa has been interpreted controversially: *Gonioctena arctica* Mannerheim, 1853 from Alaska, *Gonioctena decaspilota* (Achard, 1924) from the Scandinavian Peninsula, *Gonioctena dinah* (Bechyně, 1948) from Siberia, *Gonioctena nivosa* (Suffrian, 1851) from the Alps and *Gonioctena salicis* Motschulsky, 1860 from Transbaikalia.

In the present study, we define and review the *Gonioctena nivosa* species-group of the subgenus *Gonioctena* s. str. Six species including two new species are recognized by the following characters: apical antennomere more than twice longer than wide; first tarsomere of fore legs in male swollen; apical process of aedeagus narrow, with apex rather truncate in dorsal view, apical process pointed and slightly bent downward at apex in lateral view. We have attempted to solve its taxonomic problems based on the external morphology, geographic variation of male genitalia, coloration and distribution. Biological information on host plant and ovoviviparity is also provided.

## Material and methods

Specimens were examined with a Nikon SMZ800 microscope. Male genitalia were dissected from adult specimens softened in the closed Petri dish with wet tissue paper for 12–24 hours, cleared in 10% sodium hydroxide solution, and rinsed in distilled water. Photographs were taken by a Nikon D5200 digital camera attached to a Nikon SMZ1500 microscope, and were edited by Helicon Focus 5.3.12 and Adobe Photoshop CS5. A double slash (//) in the collecting data separates the data on different labels. Type localities are cited in the original spelling. Specimens examined in the study are deposited in the following collections:

ABC
 Andrzej O. Bieńkowski Collection, Moscow, Russia 


AWC
 Andrzej Warchałowski Collection, Wrocław, Poland 


BMNH
 The National History Museum, London, UK 


CNCI
 Canadian National Collection of Insect, Ottawa, Canada 


DBET
 Department of Biodiversity and Evolutionary Taxonomy, University of Wrocław, Wrocław, Poland 


ELEU
 Entomological Laboratory, Ehime University, Matsuyama, Japan 


ELKU
 Entomological Laboratory, Kyushu University, Fukuoka, Japan 


FKC
 František Kantner Collection, České Budějovice, Czech Republic 


HCC
 Hee-Wook Cho Collection, Andong, South Korea 


JBC
 Jan Bezděk Collection, Brno, Czech Republic 


LMC
 Lev N. Medvedev Collection, Moscow, Russia 


MLUH
 Institut für Zoologie, Martin-Luther-Universität Halle-Wittenberg, Halle, Germany 


MNHN
Museum National d’Historie Naturelle, Paris, France 


MSNM
Museo Civico di Storia Naturale, Milano, Italy 


MZHF
Finnish Museum of Natural History, University of Helsinki, Finland 


NHMB
 Naturhistorisches Museum Basel, Basel, Switzerland 


NHRS
Naturhistoriska Riksmuseet, Stockholm, Sweden 


NMPC
 Národní Muzeum, Prague, Czech Republic 


SDEI
Senckenberg Deutsches Entomologisches Institut, Müncheberg, Germany 


SEHU
 Systematic Entomology, Hokkaido University Museum, Sapporo, Japan 


TARI
Taiwan Agricultural Research Institute, Applied Zoology Division, Wufeng, Taiwan 


TLMF
 Horst Kippenberg Collection, Tiroler Landesmuseum Ferdinandeum, Innsbruck, Austria 


UZIU
Zoological Museum, Uppsala University, Uppsala, Sweden 


ZIN
Zoological Institute, Russian Academy of Sciences, St. Petersburg, Russia 


ZMHB
Museum für Naturkunde der Humboldt-Universität, Berlin, Germany 


ZMUC
Zoological Museum, University of Copenhagen, Copenhagen, Denmark 


## Taxonomy

### *Gonioctena nivosa* species-group

**Diagnosis.** Body length 4.05–7.00 mm. Males usually with much longer antennae than females; antennae in male reaching elytral humeri, as long as or longer than half length of body (Figs 1, 3, 5, 7, 9, 11); in female reaching elytral humeri or not; apical antennomere in both sexes more than twice longer than wide (Figs 13, 17, 26, 29, 55, 63). First tarsomere of fore legs in male swollen, almost as wide as or wider than third; in female not modified. Aedeagus parallel-sided or moderately narrowed apically, with apical process narrow, apex rather truncate in dorsal view; moderately or strongly curved, with apical process pointed and slightly bent downward at apex in lateral view (Figs 14, 18, 27, 30, 56, 64).

**Figures 1–6.**
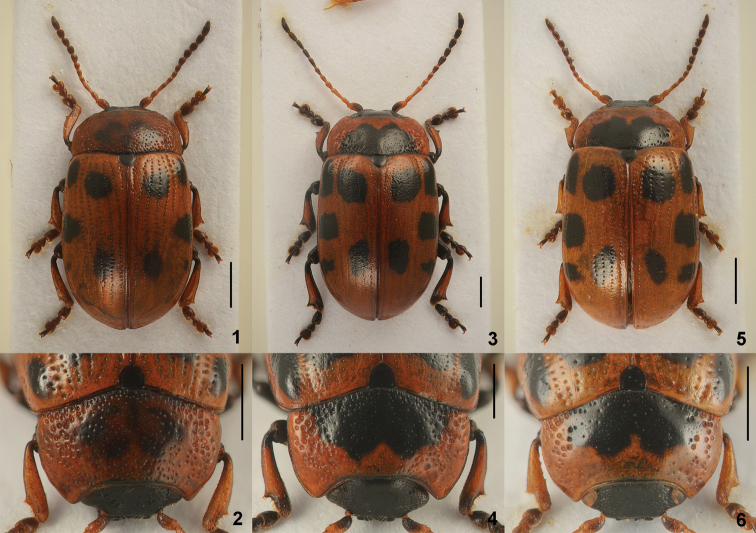
Dorsal habitus and pronotum. **1–2** *Gonioctena amurensis* sp. n., holotype **3–4** *Gonioctena gracilicornis* **5–6** *Gonioctena jani* sp. n., holotype. Scale bars = 1.0 mm.

**Figures 7–12.**
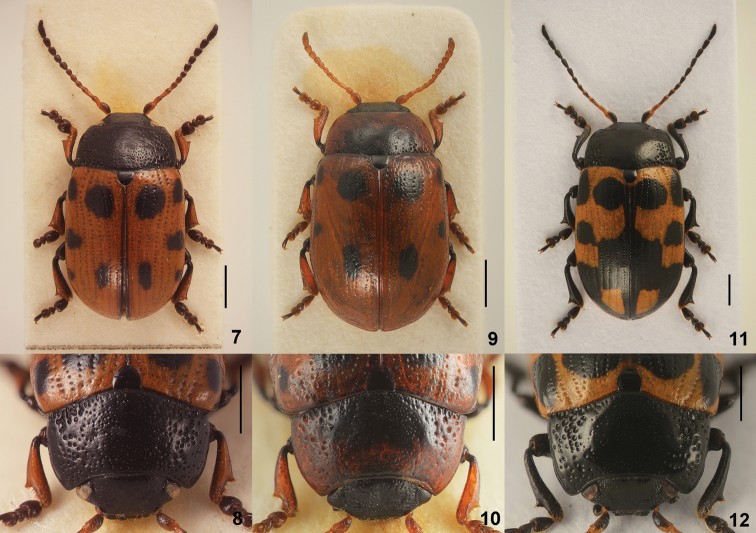
Dorsal habitus and pronotum. **7–8** *Gonioctena nivosa* **9–10** *Gonioctena norvegica*, syntype **11–12** *Gonioctena springlovae*. Scale bars = 1.0 mm.

**Figures 13–15.**
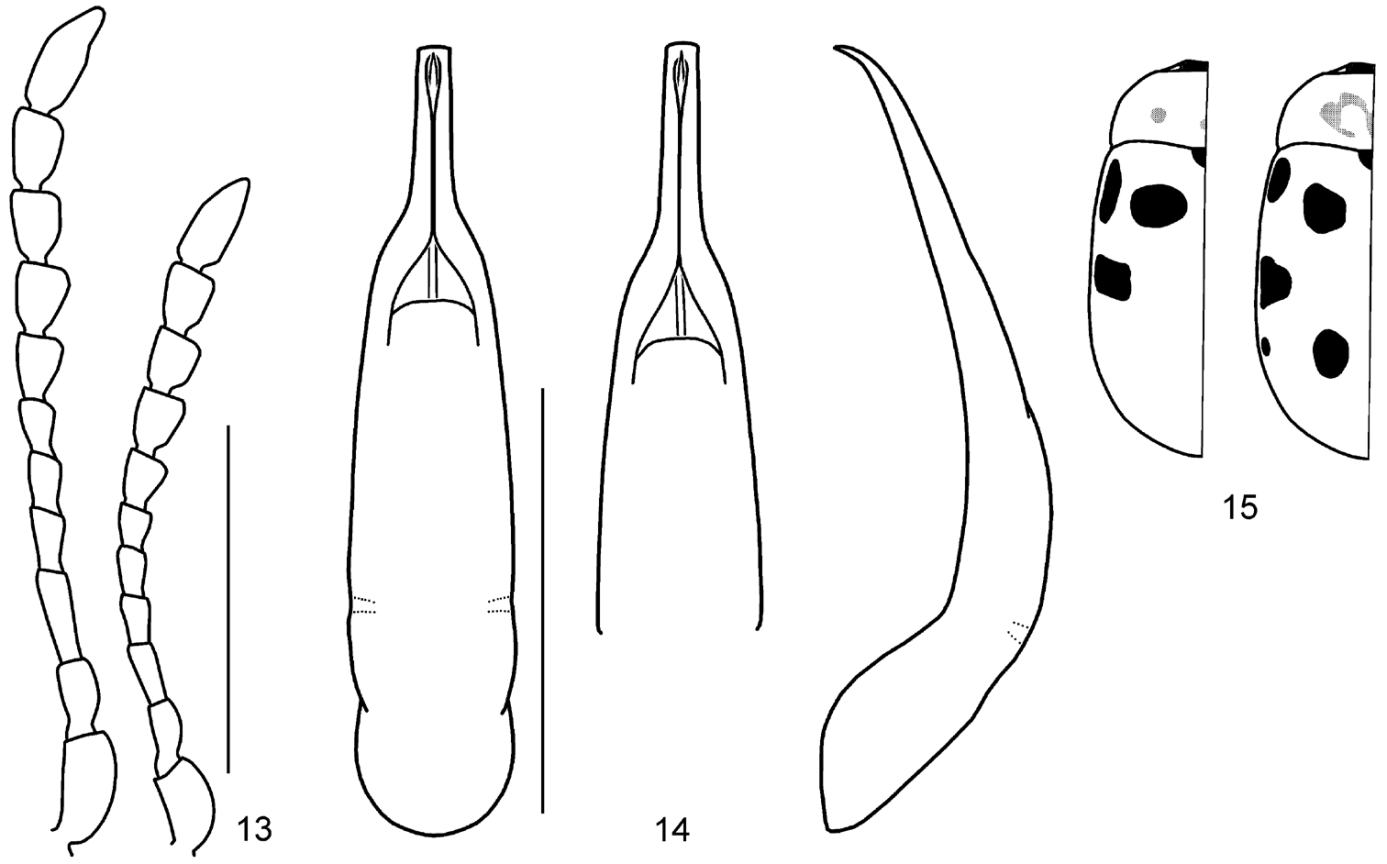
*Gonioctena amurensis* sp. n. **13** Antenna (♂, ♀) **14** Aedeagus (dorsal, apical and lateral views) **15** Color variation. Scale bars = 1.0 mm.

### Key to species of *Gonioctena nivosa* species-group

**Table:** 

1	Antennae as long as or longer than half length of body in male (Figs 1, 3, 5, 7, 11), almost or fully reaching elytral humeri in female	**2**
–	Antennae much shorter than half length of body in male (Fig. 9), not reaching elytral humeri in female. From Norway to Mongolia	***norvegica* (Strand)**
2	Antennae as long as half length of body in male (Figs 1, 5), almost reaching elytral humeri in female	**3**
–	Antennae much longer than half length of body in male (Figs 3, 7, 11), fully reaching elytral humeri in female	**4**
3	Pronotum with obscure black spots or marking (Fig. 15); aedeagus rather thin with apical process long, very slightly tapered apically (Fig. 14). Mongolia, Russia (Far East)	***amurensis* Cho & Borowiec, sp. n.**
–	Pronotum with distinct black spots or marking (Fig. 28); aedeagus rather thick with apical process short, very slightly widened apically (Fig. 27). Russia (East Siberia, Far East)	***jani* Cho & Borowiec, sp. n.**
4	Smaller, body length 4.05–6.00 mm; first tarsomere of all legs in male much strongly swollen (Fig. 7); aedeagus strongly curved in lateral view (Fig. 30). Transholarctic	***nivosa* (Suffrian)**
–	Larger, body length 5.70–7.00 mm; first tarsomere of all legs in male less strongly swollen (Figs 3, 11); aedeagus moderately curved in lateral view (Figs 18, 64)	**5**
5	Pronotum feebly rounded laterally (Fig. 12); aedeagus thin (Fig. 64). Sakhalin, Hokkaido	***springlovae* (Bechyně)**
–	Pronotum strongly rounded laterally (Fig. 4); aedeagus rather thick (Fig. 18). Russia (East Siberia, Far East, Sakhalin), Mongolia, China (Heilongjiang), Korea	***gracilicornis* (Kraatz)**

### Gonioctena (Gonioctena) amurensis

Taxon classificationAnimaliaColeopteraChrysomelidae

Cho & Borowiec sp. n.

http://zoobank.org/A9B743E2-DDE4-4F4B-A8AF-26C9A3B74D0F

**Figure 16.**
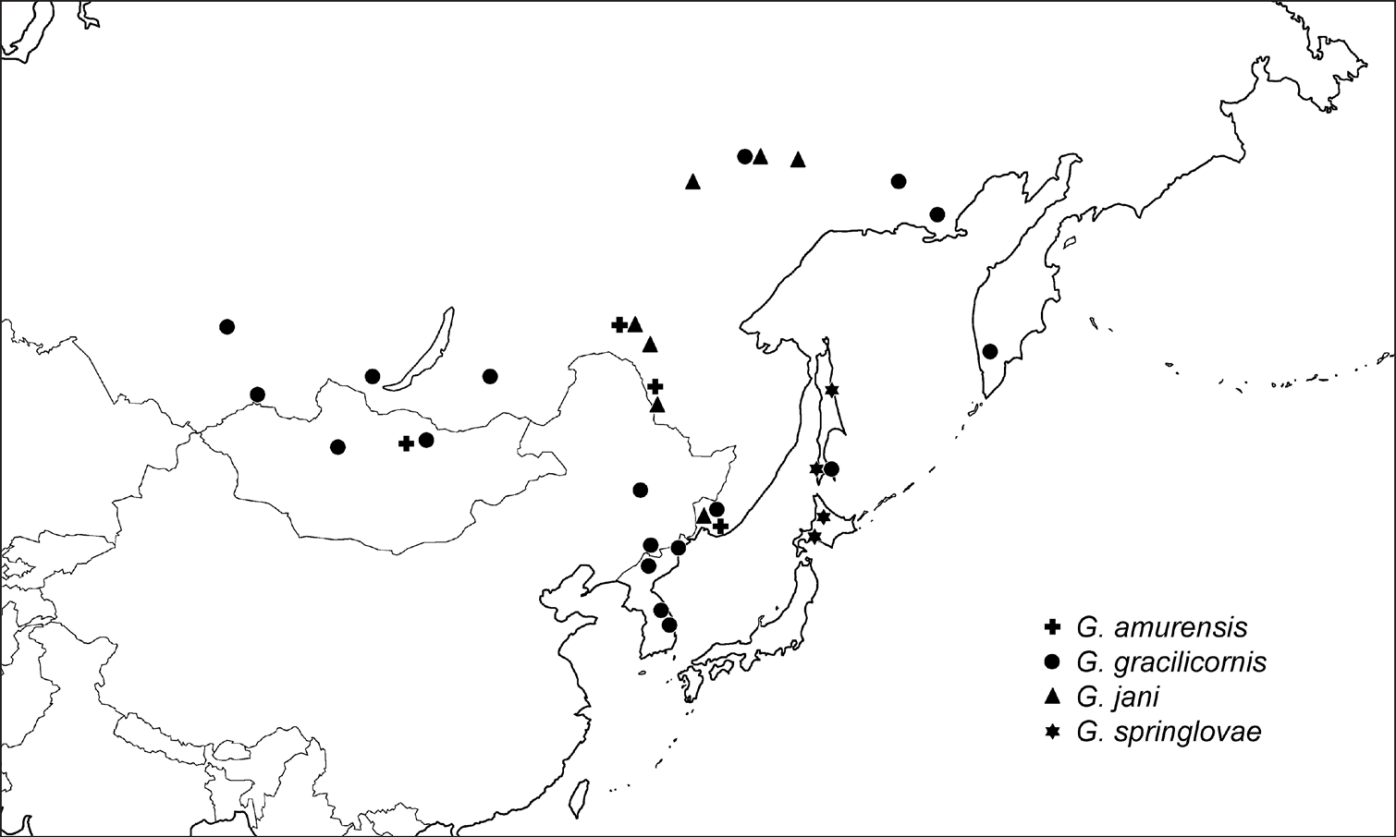
Distribution of *Gonioctena amurensis*, *Gonioctena gracilicornis*, *Gonioctena jani* and *Gonioctena springlovae* based on specimens in Asia.

Figs 1–2 , 13–15 , 16

#### Type material.

Holotype: ♂ (ZIN), Russia, Amur Oblast, Svobodnensky District, between the Malaya Pera and Bolshoy Ergel Rivers, 2.VII.1958, Zinoviev leg. // HOLOTYPUS Gonioctena (Gonioctena) amurensis sp. n. Cho & Borowiec 2015. Paratypes: 5♂♂ (ZIN), same data as holotype; 1♂, 1♀ (ZIN), Russia, Amur Oblast, Svobodnensky District, Klimoutsy Village, 40 km W Svobodny City, 14.VII.1957, Zinoviev leg.; 1♂ (ZIN), Russia, Amur Oblast, Tyndinsky District, between Djeltulak and Sosnovaya, 30.VII.1928, Obolenskiy leg.; 1♂ (NHMB), Russia, Primorsky Krai, Ussuriysk Reserve, VI.1956, L.N. Medvedev; 1♂ (TLMF), Russia, Primorsky Krai, Khasansky District, Kedrovaya Pad Nature Reserve, 1956, L. Medvedev; 1♀ (NHMB), Mongolia, Central Aimak, 21.VI.1974, V. Janovsky leg. Each paratype specimen has a type label: PARATYPUS Gonioctena (Gonioctena) amurensis sp. n. Cho and Borowiec 2015.

#### Diagnosis.

*Gonioctena amurensis* sp. n. is closely related to *Gonioctena jani* sp. n. in having small body size and similar length of antennae, however it can be distinguished by pronotum with small and moderately dense punctures on median region and large and dense punctures on lateral region (sparse punctures on median region and moderately dense punctures on lateral region in *Gonioctena jani* sp. n.) and rather thin aedeagus with relatively long apical process (rather thick with relatively short apical process in *Gonioctena jani* sp. n.).

#### Description.

Measurements in mm (n = 5): length of body: 5.00–5.50 (mean 5.16); width of body: 2.90–3.25 (mean 3.09); height of body: 1.85–2.30 (mean 2.09); width of head: 1.40–1.45 (mean 1.41); interocular distance: 0.95–1.05 (mean 0.99); width of apex of pronotum: 1.50–1.65 (mean 1.55); width of base of pronotum: 2.45–2.60 (mean 2.51); maximum width of pronotum: 2.52–2.62 (mean 2.56); length of pronotum along midline: 1.30–1.40 (mean 1.32); length of elytra along suture: 3.65–4.10 (mean 3.85).

Body oblong oval and moderately convex (Fig. 1). Head black. Mandibles black, with reddish brown band near apex. Maxillary palps reddish brown or dark brown, with apical palpomere blackish brown. Antennomeres 1–7 yellowish brown, 1 and 7 darkened, 8–11 dark brown to blackish brown. Pronotum reddish brown, with 2–3 obscure spots or an obscure marking (Fig. 15). Scutellum black. Elytra reddish brown, with 3–5 pairs of black spots. Venter black, with hypomera and apical margin of last abdominal ventrite reddish brown. Legs black, with tibiae except base and tarsi reddish brown.

*Head*. Vertex weakly convex, covered with coarse and dense punctures. Frontal suture V-shaped, coronal suture absent or weak. Frons flat, strongly depressed anteriorly, covered with moderately dense punctures. Clypeus narrow and trapezoidal. Anterior margin of labrum distinctly concave. Mandibles with 2 sharp apical teeth and a deep excavation for apical maxillary palpomere at outer side. Maxillary palps 4-segmented, with apical palpomere distinctly widened, truncate apically in male; slightly widened in female. Antennae in male as long as half length of body; antennomere 1 robust; antennomere 2 shorter than 3; antennomere 3 longer than 4; antennomeres 7–11 each distinctly longer than wide; antennomere 11 longest, about 2.48 times as long as wide (Fig. 13). Antennae in female almost reaching elytral humeri; antennomere 11 about 2.38 times as long as wide.

*Pronotum*. Lateral sides widest near base, roundly moderately narrowed anteriorly, anterior angles strongly produced (Fig. 2). Anterior and lateral margins bordered, lateral margins invisible in dorsal view. Trichobothria present on posterior angles. Disc covered with moderately dense punctures; lateral sides covered with much coarser and denser punctures, becoming larger toward base, partially confluent near basal margin; interspaces covered with fine and sparse punctures. Scutellum slightly wider than long, narrowed posteriorly.

*Elytra*. Lateral sides moderately widened posteriorly, widest beyond middle, thence roundly narrowed posteriorly. Humeral calli well developed. Disc covered with 11 regular rows of large punctures, including a short scutellar row; punctures rather irregular between 6th and 8th striae in apical half; interspaces shagreened, covered with fine and sparse punctures. Epipleura wholly visible in lateral view. Hind wings well developed.

*Venter*. Hypomera weakly rugose, with a few punctures near anterolateral corners of prosternum. Prosternum covered with coarse and dense punctures bearing long setae; prosternal process enlarged apically, bordered laterally, with moderately dense punctures. Metasternum covered with small and moderately dense punctures in median region, large and dense punctures in lateral region. Abdominal ventrites covered with dense punctures bearing short setae.

*Legs*. Moderately robust. Tibiae widened apically; fore tibia with a blunt tooth-like projection; mid and hind tibiae each with a tooth-like projection. Fore legs with tarsomere 1 strongly enlarged, distinctly wider than 3 in male; slightly narrower than 3 in female. Tarsal claws appendiculate.

*Genitalia*. Aedeagus moderately narrowed apically, with apical process rather long, very slightly tapered apically, apex truncate in dorsal view; moderately curved, with apical process pointed and slightly bent downward at apex in lateral view (Fig. 14). Spermatheca absent.

#### Etymology.

Named after the type locality, Amur region.

#### Distribution.

Mongolia, Russia (Far East) (Fig. 16).

### Gonioctena (Gonioctena) gracilicornis

Taxon classificationAnimaliaColeopteraChrysomelidae

(Kraatz, 1879)

Figs 3–4 , 16 , 17–25 , 66

Phytodecta gracilicornis Kraatz, 1879b: 135 (type locality: Amur); Weise 1893: 1129; Jacobson 1901: 128; Bechyně 1948: 115.
Gonioctena gracilicornis : Marseul 1888: 37; L. N. Medvedev 1968: 76; Jolivet 1973: 266; L. N. Medvedev and Voronova 1976: 228; L. N. Medvedev and Korotyaev 1980: 86; L. N. Medvedev and Zaytsev 1980: 105 (larva); L. N. Medvedev and Roginskaya 1988: 100 (host plant); Dubeshko and L. N. Medvedev 1989: 132 (biology); Li 1992: 184; Mikhailov and Hayashi 2000: 82.
Phytodecta (Phytodecta) gracilicornis : Weise 1916: 177; Winkler 1930: 1295; Chen 1935: 127, 1936: 86; Chûjô 1941: 74.
Gonioctena (Gonioctena) gracilicornis : Gressitt and Kimoto 1963: 358, 361; L. N. Medvedev and Zaytsev 1978: 119 (larva); L. N. Medvedev 1982: 92, 179, 252 (incl. larva); Takizawa 1985: 9; L. N. Medvedev 1992: 575; L. N. Medvedev and Dubeshko 1992: 118; V. L. Medvedev 1999: 14; Lee and An 2001: 102; V. L. Medvedev 2004: 41; Lopatin et al. 2004: 122; L. N. Medvedev 2006a: 139; Cho and Lee 2008: 105, 107; Zaytsev and L. N. Medvedev 2009: 145 (larva); Cho and Lee 2010: 58; Kippenberg 2010: 433; Warchałowski 2010: 559.
Phytodecta (Phytodecta) gracilicornis var. kiberi Chûjô, 1941: 74 (type locality: Korea, Keiki-Do, Hosen-Gun, Mt. Syoyo-Zan); Gressitt and Kimoto 1963: 362 (as synonym of Gonioctena gracilicornis).
Phytodecta (Phytodecta) gracilicornis var. munaguro Chûjô, 1941: 75 (type locality: Korea, Kankyo-Hokudo, Mt. Kwambo-Zan); Gressitt and Kimoto 1963: 362 (as synonym of Gonioctena gracilicornis).
Phytodecta (Phytodecta) gracilicornis var. signaticollis Chûjô, 1941: 75 (type locality: E Siberia); Gressitt and Kimoto 1963: 361 (as synonym of Gonioctena gracilicornis).
Gonioctena sunkangensis Kimoto & Kawase, 1966: 44 (type locality: Manchuria, Laoheishan); L. N. Medvedev 1982: 252 (as synonym of Gonioctena gracilicornis).
Gonioctena sungkangensis [sic!]: Takizawa 1985: 7.
Gonioctena (Gonioctena) sungkangensis [sic!]: Takizawa 1985: 9; Lee and An 2001: 103.
Gonioctena (Gonioctena) sunkangensis : V. L. Medvedev 2004: 41; Kippenberg 2010: 434; Yang et al. 2014: 368, 2015: 50.
Gonioctena (Gonioctena) coreana : L. N. Medvedev 1992: 573 (part) (misidentification).
Gonioctena springlovae : Li 1992: 189 (misidentification).

#### Type material.

*Gonioctena gracilicornis*: Syntypes 1♂ (SDEI), Amur // Coll. Kraatz // Dtsch Ent. Inst. Eberswalde // Lectotypus *Gonioctena gracilicornis* Kz.; 1♂ (SDEI), Amur // Paralectotypus // Coll. Kraatz // Dtsch Ent. Inst. Eberswalde; 2♂♂, 5♀♀ (SDEI), Amur // Paralectotypus // Coll. Kraatz // *Phytodecta gracilicornis* Kr. // Dtsch Ent. Inst. Eberswalde; 1♂ (SDEI), Amur, Christoph 77 // Paralectotypus // *Phytodecta gracilicornis* Kr. // Dtsch Ent. Inst. Eberswalde; 1♂ (BMNH), Cotype // Amur // Brit. Mus, 1937-250 // *Phytodecta gracilicornis* Kr. // Coll. Kraatz // Typus.

Phytodecta gracilicornis var. kiberi: Holotype in TARI.

Phytodecta gracilicornis var. munaguro: Holotype and paratype in TARI.

Phytodecta gracilicornis var. signaticollis: Type depository unknown.

*Gonioctena sunkangensis*: Holotype ♂ (ELKU), Manchuria, Laoheishan, 17.X.1918 // *Gonioctena sunkangensis* Kimoto & Kawase // HOLOTYPE.

#### Other material.

**Russia**: 1♂ (NHMB), Vladivostok, Russia, 1933, N. Filippov; 1♂ (NHMB), Russia, Primorsky Krai, Ussuriysk Reserve, VI.1956, L.N. Medvedev; 1♂ (NHMB), Magadanska oblast, 13 km N of Klepka, 27.VI.1975 // *Salix*; 1♂ (NHMB), pr. Kamenushka 30 km E Ussurijsk, 20–25.VI.1990 // USSR Ussuri, Maritime Terr., S. Kasantsev; 1♀ (NHMB), Transbaikal; 1♂ (NHMB), Tschita, Transbaikalien. Hermann Frieb.; 1♂ (NHMB), Sutschan, Ussuri; 1♂ (NHMB), Sibiria orient., Sotka-Sora, B. v.Bodemeyer; 2♂♂, 1♀ (JBC), Russia, Krasnojarskij K. Sajanogorsk, Maina, 3–9.VII.1994, leg. Kletecka; 1♂ (ABC), Russia, Primorskiy Kray, Zarechnoye 10 km SE, Ussuriysk, 43.37N 132.18E, 11.VI.1993, 200 m, leg. L. Zerche; 3♂♂, 1♀ (ABC), Russia, Tuva, S. Slopes of E. Tanu-Ola Mts., envir. Samagaltai vill., 1400–1800 m, 21.V.–11.VI.2002, Vashchenko leg.; 3♂♂ (HCC), Russia, NE Siberia, Yakutia reg., Khandyga, VII.1993, Maglis leg.; 7♂♂, 2♀♀ (HCC), Russia, S Siberia, Tuva, S slope of E Tannu Ola Mts, Samag altai v., 1600m, 10.VI.2004, S. Vaschenko leg.; 2♂♂, 1♀ (ELEU), Far East Russia, nr. Anisimovka, Primor Territ., 3.VII.1999, Y. Notsu leg.; 1♀ (ELEU), Russia, Bistraya River, Kamchatka (53.55N, 157.42E), 16.VIII.2000, T. Yamamoto leg.; 16♂♂, 5♀♀ (FKC), Russia, Primorskij kr., Arsenev env., VI.1991, leg. M. Štrba; 10♂♂, 5♀♀ (FKC), Russia, Krasnojarskij kr., Sajangorsk, Maina, 3.VII.1994, leg. Z. Kletecka; 1♂ (LMC), Saghalien, Toyohara, 16.VII.1922, Teiso Esaki; 1♂ (SDEI), Russia: Primorsky Kray, Sikhote-Alin, Biof. Stat. 35 km SE, Chuguyevka // 44.05N 134.02E, 31.V.1993, 650 m, leg. L. Zerche et al.; 1♂ (SDEI), Russia, Primorsky Kray, Krounovka, Medveditsa river, 40 km SW Ussuriysk, 250 m // 43°3’N, 131°15’E, 2–6.VIII.1993, leg. E.K. Groll; 1♂ (SDEI), Amur, Christoph 77 // Coll. Kraatz; 1♂, 1♀ (ZIN), Russia, Magadan Oblast, Tenkinsky District, Kolyma River, Duskanya Village (or river outlet), 8.VIII.1979, Migovich leg.; 1♂ (ZIN), Russia, Magadan Oblast, Tenkinsky District, Kolyma River, Duskanya Village (or river outlet), 3.VIII.1979, Russ leg.; 1♂ (ZIN), Russia, Primorsky Krai, Ussuriysky Urban Okrug, Kaimanovka Village (Suputinsky Dok Village), 14.VI.1960, Kabakov leg.; 1♂, 1♀ (MNHN), Museum Paris Siberie env. D’Irkoutsk, Nilova Poustine, D. Busson 1913; 1♀ (NMPC), Transbaikalien, Leder Reitter // Collectio A. Fleischer // Ovoviviparous, Det. H.W. Cho; 1♂ (TLMF), Kamchatka, Elisovo (53°20’N, 158°25’E), 13.VIII.1995, leg. S. Bohl; 2♂♂ (TLMF), VII.–VIII., Russia, Primorsky Krai, Kedrovaja pad, leg. + det. L. Medvedev; 2♂♂, 2♀♀ (TLMF), Transbaicalia, Selenga-Tal; 1♂ (TLMF), Blagovetchensk, leg. Zaizev; 3♂♂, 3♀♀ (TLMF), Russia, Tuva, Shuurmag, Khorumnug-Tayga, 800m, 29.VI.–1.VII.1998, leg. Vashchenko; 7♂♂, 10♀♀ (TLMF), E-Sibiria, Chabarowsk, Ochotsk surr., Ulia-river, 13.VII.–7.VIII.1985, leg. Ryvkin & Veselova; 1♂ (TLMF), Russia, Amur reg., Selemdgin distr., Tamsche, 4.IX.2004, leg. Ryvkin; 1♀ (TLMF), Russia, Amur reg., Selemdgin distr., Norsk vill., 2.VIII.2004, leg. Ryvkin & Veselova. **Mongolia**: 2♀♀ (FKC), Mongolia, 50 km E of Ulanbatar, Tuul riv., 22.VI.2003, J. Halada lgt.; 1♂, 1♀ (NMPC), Nordl. Mongolei. Changai, Leder. // Coll. Achard Mus. Pragense; 1♂ (NMPC), Mongolia, Reitter // Coll. Achard Mus. Pragense. **China**: 1♂ (SDEI), China, Charbin, v. Bennigsen // Fleischer det.; 1♂ (SDEI), Erzendjanzsy, Manshukuo, leg. W. Alin, 21.VI.1940; 1♂ (SDEI), Erzendjanzsy, Manshukuo, leg. W. Alin, 15.VI.1941; 2♂♂ (MNHN), Museum Paris Mandjourie Ourga a Tsitsikhar, J. Chaffnjon 174-96; 1♂ (NMPC), Charbin v. Bennigsen // Collectio A. Fleischer // Phytodecta gracilicornis ab. innocens Mader 1945 Det. J. Bechyně. **North Korea**: 1♂ (NHMB), PuRyong, N. Korea; 1♂, 1♀ (SEHU), Rangrim, Nth Korea, 1.VII.1980. **South Korea**: 3♂♂ (HCC), Korea, Gyeongbuk Prov., Bonghwa-gun, Socheon-myeon, Buncheon-ri, 13.V.2006, H.W. Cho; 7♂♂, 1♀ (HCC), Korea, Gangwon Prov., Pyeongchang-gun, Mt. Gyebangsan, 30.V.2006, H.W. Cho; 3♂♂, 1♀ (HCC), Korea, Gangwon Prov., Pyeongchang-gun, Mt. Odaesan, 30.V.2006, H.W. Cho; 1♂ (HCC), Korea, Gangwon Prov., Pyeongchang-gun, Mt. Odaesan, 6.VI.2009, H.W. Cho; 1♂ (HCC), Korea, Gangwon Prov., Hongcheon-gun, Nae-myeon, Myeonggae-ri, 13.VII.2002, D.Y. Lee; 1♂ (HCC), Korea, Gangwon Prov., Hongcheon-gun, Nae-myeon, Unduryeong, 11.VI.1997, S.B. Ahn.

#### Diagnosis.

This species is very similar to *Gonioctena springlovae* in having large body size, long antennae and similar shape of aedeagus. However, *Gonioctena gracilicornis* can be distinguished by pronotum with strongly rounded lateral sides (feebly rounded in *Gonioctena springlovae*), pronotum reddish brown, with or with a large black marking, sometimes entirely black (always entirely black in *Gonioctena springlovae*) and aedeagus rather thick (thin in *Gonioctena springlovae*).

#### Redescription.

Measurements in mm (n = 5): length of body: 6.20–7.00 (mean 6.66); width of body: 3.70–4.20 (mean 3.99); height of body: 2.60–3.20 (mean 2.87); width of head: 1.77–1.95 (mean 1.84); interocular distance: 1.17–1.30 (mean 1.24); width of apex of pronotum: 2.00–2.30 (mean 2.13); width of base of pronotum: 3.02–3.40 (mean 3.22); maximum width of pronotum: 3.10–3.47 (mean 3.28); length of pronotum along midline: 1.57–1.70 (mean 1.63); length of elytra along suture: 4.60–5.35 (mean 5.03).

Body oblong oval and moderately convex (Fig. 3). Coloration extremely variable. Head black, with reddish brown band near apex of mandibles. Antennomeres 1–5 yellowish brown, sometimes darkened, 6–7 dark brown to blackish brown, 8–11 black. Pronotum reddish brown, with or without a large black marking, sometimes entirely black (Fig. 25). Scutellum black. Elytra reddish brown, with or without 5 pairs of black spots, sometimes enlarged and connected with each other. Venter black, with hypomera and apical margin of last abdominal ventrite reddish brown. Legs black, with tibiae reddish brown except base and inner margin and tarsi dark brown to blackish brown, sometimes tibiae and tarsi largely black. Rarely body almost completely black except antennae.

**Figures 17–25.**
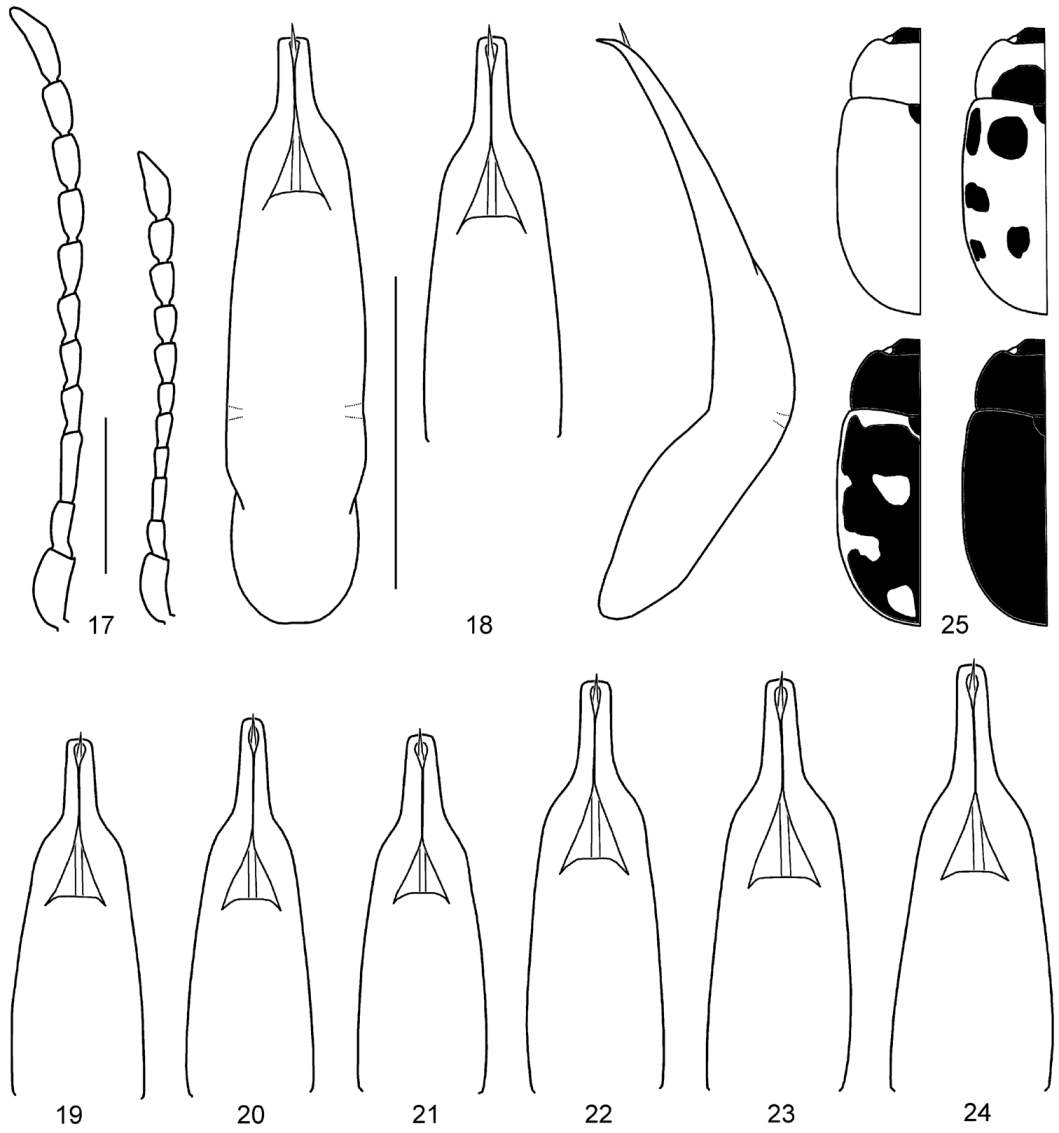
*Gonioctena gracilicornis*. **17** Antenna (♂, ♀) **18** Aedeagus (Amur) **19** Aedeagus (Tuva, Russia) **20** Aedeagus (Ulan Bator, Mongolia) **21** Aedeagus (Transbaikalia) **22** Aedeagus (Charbin, China) **23** Aedeagus (Pyeongchang, South Korea) **24** Aedeagus (Anisimovka, Russia) **25** Color variation. Scale bars = 1.0 mm.

*Head*. Vertex weakly convex, covered with sparse punctures, becoming denser toward sides. Frontal suture V-shaped, coronal suture weak. Frons flat, strongly depressed anteriorly, covered with moderately dense punctures. Clypeus very narrow and trapezoidal. Anterior margin of labrum distinctly concave. Mandibles with 2 sharp apical teeth and a deep excavation for apical maxillary palpomere at outer side. Maxillary palps 4-segmented, with apical palpomere distinctly widened, truncate apically in male; slightly widened in female. Antennae in male longer than half length of body; antennomere 1 robust; antennomere 2 shorter than 3; antennomere 3 longer than 4; antennomeres 7–11 elongate; antennomere 11 longest, about 3.44 times as long as wide (Fig. 17). Antennae in female reaching elytral humeri; antennomere 11 about 2.72 times as long as wide.

*Pronotum*. Lateral sides widest near base, roundly moderately narrowed anteriorly, anterior angles strongly produced (Fig. 4). Anterior and lateral margins bordered, lateral margins well visible in dorsal view. Trichobothria present on posterior angles. Disc covered with sparse punctures; lateral sides covered with much coarser and denser punctures, becoming larger toward base, partially confluent near basal margin; interspaces covered with fine and sparse punctures. Scutellum variable in length, as long as wide, longer than wide or wider than long.

*Elytra*. Lateral sides slightly widened posteriorly, widest beyond middle, thence roundly narrowed posteriorly. Humeral calli well developed. Disc covered with 11 regular rows of large punctures, including a short scutellar row; interspaces shagreened in some specimens, covered with fine and sparse punctures. Epipleura wholly visible in lateral view. Hind wings well developed.

*Venter*. Hypomera weakly rugose, with dense punctures on anterior side. Prosternum covered with coarse and dense punctures bearing long setae; prosternal process enlarged apically, bordered laterally, with sparse punctures. Metasternum covered with small and sparse punctures in median region, large and dense punctures in lateral region. Abdominal ventrites covered with dense punctures bearing short setae.

*Legs*. Moderately robust. Tibiae widened apically, with a tooth-like projection. Fore legs with tarsomere 1 enlarged, slightly wider or narrower than 3 in male; distinctly narrower than 3 in female. Tarsal claws appendiculate.

*Genitalia*. Aedeagus rather thick, moderately narrowed apically, with apical process rather thick in dorsal view; moderately curved, with apical process pointed and slightly bent downward at apex in lateral view (Figs 18–24). Spermatheca absent.

#### Distribution.

Russia (East Siberia, Far East, Sakhalin), Mongolia, China (Heilongjiang), North Korea, South Korea (Fig. 16).

#### Host plant.

Salicaceae: *Salix caprea*, *Salix rorida*, *Salix sachalinensis* (L. N. Medvedev 1968); *Salix* spp. (L. N. Medvedev and Zaytsev 1978, L. N. Medvedev 1982, 1992, L. N. Medvedev and Roginskaya 1988, L. N. Medvedev and Dubeshko 1992, Zaytsev and L. N. Medvedev 2009).

#### Remarks.

*Gonioctena gracilicornis* is widely distributed in the Northeastern Palearctic region (Fig. 16) and is slightly variable in the shape of aedeagus (Figs 18–24). Gonioctena gracilicornis var. kiberi, *munaguro*, *signaticollis* were described by Chûjô (1941) and synonymized with *Gonioctena gracilicornis* by Gressitt and Kimoto (1963). However, the type specimens of these variations have not been examined and their taxonomic status needs to be re-examined. Medvedev (1982) synonymized *Gonioctena sunkangensis* with *Gonioctena gracilicornis*, however he did not examine the type of *Gonioctena sunkangensis*. We examined types of both species and confirm that both are conspecific. Lectotype label of *Gonioctena gracilicornis* by L. N. Medvedev has not been published, and thus invalid. Li’s record (1992) is probably based on misidentified *Gonioctena gracilicornis* because *Gonioctena springlovae* has not been recorded from China. Female laid larvae which were enclosed within chorion on leaves of *Salix* sp. in South Korea, therefore this species is ovoviviparous (Fig. 66).

### Gonioctena (Gonioctena) jani

Taxon classificationAnimaliaColeopteraChrysomelidae

Cho & Borowiec sp. n.

http://zoobank.org/63AE87F3-E9F9-4481-812C-EC824EA4DFFF

**Figures 26–28.**
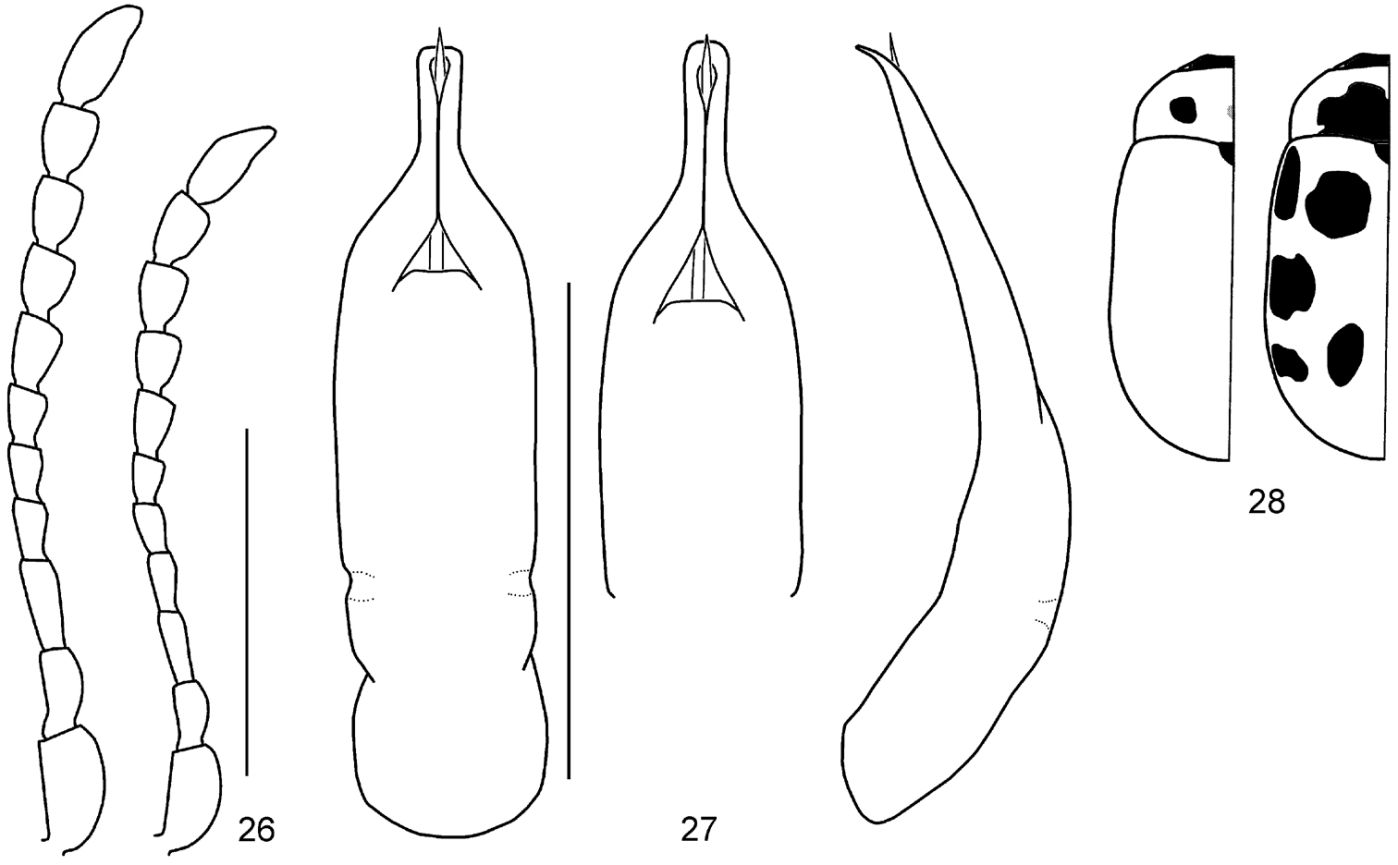
*Gonioctena jani* sp. n. **26** Antenna (♂, ♀) **27** Aedeagus **28** Color variation. Scale bars = 1.0 mm.

Figs 5–6 , 16 , 26–28

#### Type material.

Holotype: ♂ (ZIN), Russia, Sakha Republic, Amginsky District, Krestyah Village, 18.VII.1928, ex Museum of Yakutia // HOLOTYPUS Gonioctena (Gonioctena) jani sp. n. Cho & Borowiec 2015. Paratypes: 3♂♂, 1♀ (ZIN), same data as holotype; 2♂♂ (NHMB), Oberer Amur // ex Orig. Samlg. J. Breit Wien; 1♂ (ABC), Eastern Yakutia Republic, Suntar-Khayata range, 1290 m, on *Salix*, 8.VII.2002, O. Khruleva leg.; 1♂ (ABC), Amur Reg., Zeya Distr., Zeyskiy Reservoir, Tukurlinga ridge, 21–24.VI.2006, E.V. Guskova leg.; 1♂ (TLMF), Russia, Primorsky Krai, Khasansky District, Kedrovaya Pad Nature Reserve, VII–VIII.1956, Medvedev; 1♂ (TLMF), Russia, Amur oblast, Blagoveshchensk; 3♂♂, 9♀♀ (TLMF), Russia, Yakutia Republic, Khandyga, VII.1993, L. Naglis. Each paratype specimen has a type label: PARATYPUS Gonioctena (Gonioctena) jani sp. n. Cho & Borowiec 2015.

#### Diagnosis.

*Gonioctena jani* sp. n. is closely related to *Gonioctena amurensis* sp. n. in having small body size and similar length of antennae, however it can be distinguished by pronotum with sparse punctures on median region and moderately dense punctures on lateral region (small and moderately dense punctures on median region and large and dense punctures on lateral region in *Gonioctena amurensis* sp. n.) and aedeagus rather thick with relatively short apical process (rather thin with relatively long apical process in *Gonioctena amurensis* sp. n.).

#### Description.

Measurements in mm (n = 5): length of body: 5.00–5.70 (mean 5.30); width of body: 3.00–3.40 (mean 3.20); height of body: 2.10–2.40 (mean 2.18); width of head: 1.42–1.60 (mean 1.52); interocular distance: 1.02–1.12 (mean 1.07); width of apex of pronotum: 1.57–1.75 (mean 1.66); width of base of pronotum: 2.47–2.77 (mean 2.63); maximum width of pronotum: 1.80–2.75 (mean 2.45); length of pronotum along midline: 1.30–1.45 (mean 1.37); length of elytra along suture: 3.70–4.40 (mean 3.96).

Body oblong oval and moderately convex (Fig. 5). Head black. Mandibles black, with reddish brown band near apex. Maxillary palps reddish brown or dark brown, with apical palpomere black. Antennomeres 1–7 yellowish brown, 1 and 7 slightly darkened, 8–11 reddish brown to dark brown. Pronotum reddish brown, with 3 spots or a large marking (Fig. 28). Scutellum black. Elytra reddish brown, with or without 5 pairs of black spots. Venter black, with hypomera, apical and lateral parts of abdominal ventrites 3–5 reddish brown. Legs black, with tibiae reddish brown except base and tarsi dark brown to reddish brown.

*Head*. Vertex weakly convex, covered with coarse and dense punctures. Frontal suture V-shaped, coronal suture absent or weak. Frons flat, strongly depressed anteriorly, covered with dense punctures. Clypeus narrow and trapezoidal. Anterior margin of labrum distinctly concave. Mandibles with 2 sharp apical teeth and a deep excavation for apical maxillary palpomere at outer side. Maxillary palps 4-segmented, with apical palpomere distinctly widened, truncate apically in male; slightly widened in female. Antennae in male almost as long as half length of body; antennomere 1 robust; antennomere 2 shorter than 3; antennomere 3 longer than 4; antennomeres 7–11 each distinctly longer than wide; antennomere 11 longest, about 2.22 times as long as wide (Fig. 26). Antennae in female almost reaching elytral humeri; antennomere 11 about 2.33 times as long as wide.

*Pronotum*. Lateral sides widest near base, roundly moderately narrowed anteriorly, anterior angles strongly produced (Fig. 6). Anterior and lateral margins bordered, lateral margins invisible in dorsal view. Trichobothria present on posterior angles. Disc covered with sparse punctures; lateral sides covered with much coarser and denser punctures, becoming larger toward base, partially confluent near basal margin; interspaces covered with fine and sparse punctures. Scutellum slightly wider than long, narrowed posteriorly.

*Elytra*. Lateral sides moderately widened posteriorly, widest beyond middle, thence roundly narrowed posteriorly. Humeral calli well developed. Disc covered with 11 regular rows of large punctures, including a short scutellar row; punctures rather irregular between 6th and 8th striae in apical half; interspaces shagreened in female, covered with fine and sparse punctures. Epipleura wholly visible in lateral view. Hind wings well developed.

*Venter*. Hypomera weakly rugose, with a few punctures near anterolateral corners of prosternum. Prosternum covered with coarse and dense punctures bearing long setae; prosternal process enlarged apically, bordered laterally, with sparse punctures. Metasternum covered with small and sparse punctures in median region, large and dense punctures in lateral region. Abdominal ventrites covered with dense punctures bearing short setae.

*Legs*. Moderately robust. Tibiae widened apically, with a tooth-like projection. Fore legs with tarsomere 1 strongly enlarged, distinctly wider than 3 in male; slightly narrower than 3 in female. Tarsal claws appendiculate.

*Genitalia*. Aedeagus rather thick, parallel-sided in middle, with apical process rather short, very slightly widened apically, apex rather truncate in dorsal view; moderately curved, with apical process pointed and slightly bent downward at apex in lateral view (Fig. 27). Spermatheca absent.

#### Etymology.

Dedicated to Jan Bezděk (Brno, Czech Republic), the well-known specialist in Chrysomelidae.

#### Distribution.

Russia (East Siberia, Far East) (Fig. 16).

#### Host plant.

One specimen was collected on *Salix* sp. (Salicaceae) in Sakha Republic.

### Gonioctena (Gonioctena) nivosa

Taxon classificationAnimaliaColeopteraChrysomelidae

(Suffrian, 1851)

Figs 7–8 , 29–32 , 33–54 , 67

Chrysomela affinis Gyllenhal, 1808: 257 nec Fabricius, 1787: 67 (type locality: Lapponia); Suffrian 1851: 218 (part).
Gonioctena affinis : Chevrolat 1836: 403; Mannerheim 1852: 369; Letzner 1864: 142; Weise 1884: 500; Marseul 1888: 41; Székessy 1934: 33; L. N. Medvedev 1968: 77; L. N. Medvedev and Voronova 1976: 228; L. N. Medvedev and Korotyaev 1980: 81, 86; L. N. Medvedev and Zaytsev 1980: 104, 106 (larva); L. N. Medvedev and Roginskaya 1988: 100 (host plant); Dubeshko and L. N. Medvedev 1989: 130 (biology); Steinhausen 1996: 74 (larva).
Phytodecta affinis : Gradl 1882: 330; Kraatz 1879a: 53; Kittel 1884: 32; Weise 1893: 1130, 1906: 561; Holdhaus and Lindroth 1939: 208; Bechyně 1948: 92, 118, 124; L. N. Medvedev 1963: 114.
Phytodecta (Phytodecta) affinis : Weise 1916: 176; Winkler 1930: 1296; Chen 1935: 127; Mohr 1966: 185.
Gonioctena (Gonioctena) affinis : L. N. Medvedev and Zaytsev 1978: 119 (larva); L. N. Medvedev 1982: 91, 179, 252 (incl. larva), 1992: 575; L. N. Medvedev and Dubeshko 1992: 118; V. L. Medvedev 1999: 14; L. N. Medvedev 2006a: 139; Zaytsev and L. N. Medvedev 2009: 145 (larva).
Chrysomela nivosa Suffrian, 1851: 222 (type locality: Austria, Kärnten).
Gonioctena nivosa : Letzner 1864: 143 (biology); Marseul 1888: 45; Holdhaus and Lindroth 1939: 208 (as synonym of Gonioctena affinis); Wilcox 1972: 22; Steinhausen 1996: 75 (larva); Clark et al. 2004: 110.
Phytodecta nivosa : Kraatz 1879a: 54; Weise 1884: 500, 1891: 160, 1893: 1129; Székessy 1934: 33 (as synonym of Gonioctena affinis); Kippenberg 1994: 84.
Phytodecta nivosus : Weise 1906: 561; Achard 1924: 32; Bechyně 1948: 92, 118, 125.
Phytodecta (Phytodecta) nivosa : Reitter 1913: 129; Cantonnet 1968: 40, 43.
Phytodecta (Phytodecta) nivosus : Weise 1916: 178; Winkler 1930: 1295; Mohr 1966: 185.
Gonioctena (Gonioctena) nivosa : Daccordi et al. 1991: 96; Steinhausen 1994: 277 (larva); Warchałowski 1994: 104, 118, 2003: 311; Winkelman and Debreuil 2008: 142; Kippenberg 2010: 433; Warchałowski 2010: 559.
Chrysomela stenomera Dufour, 1851: 353 (type locality: Eaux-Bonnes); Weise 1906: 561 (as aberration of Gonioctena nivosa).
Phytodecta nivosus var. stenomera : Achard 1924: 32.
Gonioctena arctica Mannerheim, 1853: 257 (type locality: Kenai); Crotch 1873: 52; Holdhaus and Lindroth 1939: 208 (as synonym of Gonioctena affinis); Brown 1952: 340; Silfverberg 1992: 69, 1994a: 508, 1994b: 32; Mikhailov and Hayashi 2000: 82; Mikhailov 2001: 61; Silfverberg 2004: 82.
Chrysomela arctica : Suffrian 1858: 382.
Phytodecta arctica : Stål 1865: 329; Kraatz 1879a: 55, 56 (as synonym of Gonioctena nivosa); Schaeffer 1924: 140; Brown 1942: 100.
Gonioctena nivosa arctica : Wilcox 1972: 22.
Gonioctena (Gonioctena) nivosa arctica : Riley et al. 2003: 44.
Gonioctena (Gonioctena) arctica : Bieńkowski 2004: 67; Kippenberg 2010: 432; Warchałowski 2010: 559; Yang et al. 2014: 360, 2015: 49.
Gonioctena salicis Motschulsky, 1860: 223 (type locality: Daourie); Marseul 1888: 42; L. N. Medvedev 1982: 252 (as synonym of Gonioctena affinis), 2006b: 416 (as synonym of Gonioctena affinis).
Phytodecta salicis : Kraatz 1879b: 136; Jacobson 1901: 128.
Phytodecta (Phytodecta) salicis : Weise 1916: 179; Winkler 1930: 1296; Chen 1935: 128.
Phytodecta nivosa var. rufula Kraatz, 1879a: 55 (type locality: Tyrol); Weise 1884: 501; Marseul 1888: 45 (as synonym of Gonioctena nivosa).
Phytodecta nivosa var. rufulus : Achard 1924: 32.
Phytodecta affinis var. clythroides Gradl, 1882: 331 (type locality: Tirol).
Phytodecta nivosus ab. clytroides [sic!]: Weise 1916: 178 (as aberration of Gonioctena nivosa).
Phytodecta nivosus var. clytroides [sic!]: Achard 1924: 33.
Phytodecta affinis var. marginata Gradl, 1882: 331 (type locality: Tirol); Weise 1916: 178 (as aberration of Gonioctena nivosa).
Phytodecta nivosus var. marginatus : Achard 1924: 33.
Phytodecta affinis var. nana Gradl, 1882: 330 (type locality: Tirol); Weise 1916: 178 (as aberration of Gonioctena nivosa).
Phytodecta nivosus var. nanus : Achard 1924: 32.
Phytodecta affinis var. nigricollis Gradl, 1882: 331 (type locality: Tirol); Weise 1916: 178 (as aberration of Gonioctena nivosa).
Phytodecta affinis var. octopunctata Gradl, 1882: 330 (type locality: Tirol); Weise 1916: 178 (as aberration of Gonioctena nivosa).
Phytodecta nivosus var. octopunctatus : Achard 1924: 32.
Phytodecta affinis var. tyrolensis Gradl, 1882: 331 (type locality: Tirol); Weise 1916: 178 (as aberration of Gonioctena nivosa).
Phytodecta nivosus var. tyrolensis : Achard 1924: 33.
Phytodecta nivosa var. aethiops Heyden, 1883: 53 (type locality: Stilfserjoch); Weise 1906: 561 (as aberration of Gonioctena nivosa).
Phytodecta nivosus var. aethiops : Achard 1924: 33.
Phytodecta nivosa var. apicalis Heyden, 1883: 53 (type locality: Stilfserjoch); Weise 1906: 561 (as aberration of Gonioctena nivosa).
Phytodecta nivosa var. eppelsheimi Weise, 1884: 501 (type locality: Stilfser); Marseul 1888: 45 (as synonym of Gonioctena nivosa).
Phytodecta nivosus var. eppelsheimi : Achard 1924: 33.
Phytodecta nivosa var. funesta Weise, 1884: 501 (type locality: not indicated); Marseul 1888: 45 (as synonym of Gonioctena nivosa).
Phytodecta nivosa var. personata Weise, 1884: 501 (type locality: Tirol); Marseul 1888: 45 (as synonym of Gonioctena nivosa).
Phytodecta scutellaris Sahlberg, 1887: 55 nec Baly, 1862: 27 (type locality: Alaska, Porte Clarence); Brown 1942: 100 (as synonym of Gonioctena arctica); Kippenberg 2010: 437 (as nomen dubium).
Phytodecta (Phytodecta) scutellaris : Weise 1916: 181; Winkler 1930: 1296.
Phytodecta nivosa var. ruficollis Weise, 1891: 160 (type locality: Brenner); Weise 1906: 561 (as aberration of Gonioctena nivosa).
Phytodecta nivosus var. ruficollis : Achard 1924: 32.
Phytodecta linnaeana bergrothi Jacobson, 1901: 128 (type locality: Fl. Jenissej). **syn. n.**
Phytodecta (Phytodecta) linnaeanus var. bergrothi : Winkler 1930: 1295.
Phytodecta nivosa var. cedehensis Ronchetti, 1922: 89 (type locality: Monte Cevedale). **syn. n.**
Phytodecta affinis var. decaspilotus Achard, 1924: 32 (type locality: Norvège, Dowre); Winkler 1930: 1296 (as aberration of Gonioctena affinis).
Gonioctena decaspilota : Silfverberg 1977: 94.
Phytodecta decaspilota : Kippenberg 1994: 84.
Gonioctena (Gonioctena) decaspilota : Warchałowski 1994: 103, 104 (incl. larva), 2003: 311; Lopatin et al. 2004: 121; L. N. Medvedev 2014: 36.
Phytodecta affinis var. hamatus Achard, 1924: 32 (type locality: Lapponia); Winkler 1930: 1296 (as aberration of Gonioctena affinis).
Phytodecta nivosus var. immarginatus Achard, 1924: 33 (type locality: Helvetia); Winkler 1930: 1295 (as aberration of Gonioctena nivosa).
Phytodecta (Phytodecta) nivosus var. immarginatus : Chen 1936: 86.
Phytodecta (Phytodecta) nivosus immarginatus : Chen 1935: 128.
Phytodecta linnaeanus var. simplex Achard, 1924: 31 nec Suffrian, 1858: 383 (type locality: Kureika). **syn. n.**
Phytodecta linnaeanus var. mutatus Achard, 1924: 31 (replacement name for Phytodecta linnaeanus var. simplex). **syn. n.**
Phytodecta nivosa var. undulatus Pic, 1924: 27 (type locality: Alpes, Col du Pallet); Winkler 1930: 1295 (as aberration of Gonioctena nivosa).
Phytodecta dinah Bechyně, 1948: 118, 123 (type locality: Siberia).
Gonioctena (Gonioctena) dinah : Gressitt and Kimoto 1963: 358, 361; L. N. Medvedev 1992: 575 (as synonym of Gonioctena affinis); V. L. Medvedev 2004: 41; Kippenberg 2010: 433.
Phytodecta occidentalis : Bechyně 1948: 118, 124 (misidentification).
Gonioctena arctica alberta Brown, 1952: 340 (type locality: Alberta, Nordegg). **syn. n.**
Gonioctena nivosa alberta : Wilcox 1972: 22.
Gonioctena (Gonioctena) nivosa alberta : Riley et al. 2003: 44.

#### Type material.

*Chrysomela affinis*: Lectotype ♂ (UZIU), hereby designated, 168 // LECTOTYPUS *Chrysomela affinis* Gyllenhal, 1808 des. H.W. Cho 2014 // *Gonioctena nivosa* (Suffrian, 1851) det. H.W. Cho 2014. Paralectotypes: 1♂ (UZIU), 34; 1♀ (UZIU), Lappon., Schh. [= Lapponia, Schönherr]; 1♀ (UZIU), Bog. [= Carl Johan Bogeman]; 1♀ (UZIU), Lappon., Schh.; 1♀ (UZIU), *Chrysomela affinis* var., e. Lappon., Mannerheim; 2♀♀ (UZIU), gg. // Lappon., Schh.; 3♂♂, 3♀♀ (UZIU), no data; each specimen has a label, PARALECTOTYPUS *Chrysomela affinis* Gyllenhal, 1808 des. H.W. Cho 2014 // *Gonioctena nivosa* (Suffrian, 1851) det. H.W. Cho 2014. 1♀ (UZIU), 36 // *Chrysomela affinis* var., e. Lappon., Mannerheim; 1♂ (UZIU), 482 // Dej. [= Pierre F.M.A. Dejean]; 1♂, 2♀♀ (UZIU), Dej.; 1♀ (UZIU), no data; each specimen has a label, PARALECTOTYPUS *Chrysomela affinis* Gyllenhal, 1808 des. H.W. Cho 2014 // *Gonioctena linnaeana* (Schrank, 1781) det. H.W. Cho 2014.

*Chrysomela nivosa*: Lectotype ♂ (MLUH), hereby designated, 23950 (Kärnten) // MLU Halle, WB Zoologie, S.-Nr. 7/1/8 // LECTOTYPUS *Chrysomela nivosa* Suffrian, 1851 des. H.W. Cho 2014. Paralectotypes: 1♂ (MLUH), 9930 (Switzerland) // MLU Halle, WB Zoologie, S.-Nr. 7/1/8; 1♂ (MLUH), 9931 (Switzerland) // MLU Halle, WB Zoologie, S.-Nr. 7/1/8; 1♂ (MLUH), 9932 (Switzerland) // MLU Halle, WB Zoologie, S.-Nr. 7/1/8; 1♂ (MLUH), 14692 (Kärnten) // MLU Halle, WB Zoologie, S.-Nr. 7/1/8. Each paralectotype specimen has a type label: PARALECTOTYPUS *Chrysomela nivosa* Suffrian, 1851 des. H.W. Cho 2014.

*Chrysomela stenomera*: Type depository unknown.

*Gonioctena arctica*: Lectotype ♂ (MZHF), hereby designated, Kenai // Holmberg // *Gonioctena arctica* Mannerh. Kenai d.j. // LECTOTYPUS *Gonioctena arctica* Mannerheim, 1853 des. H.W. Cho 2014 // *Gonioctena nivosa* (Suffrian, 1851) det. H.W. Cho 2014. Paralectotypes: 5♂♂, 9♀♀ (MZHF), Kenai // Holmberg // PARALECTOTYPUS *Gonioctena arctica* Mannerheim, 1853 des. H.W. Cho 2014 // *Gonioctena nivosa* (Suffrian, 1851) det. H.W. Cho 2014.

*Gonioctena salicis*: Lectotype (designated by L. N. Medvedev, 2006b): ♂ (LMC), type // *Gonioctena salicis* Motsch. Sib. Armenia // Lectotypus *Gonioctena salicis* Motsch. L. Medvedev design. Paralectotypes: 2♂♂, 3♀♀ (LMC), Paralectotypus *Gonioctena salicis* Motsch. L. Medvedev design.; 1♂ (BMNH), Type Motsch. // *Gonioctena salicis* Motsch. Siberia orient. Type Motsch. Schaufuss Janson // Baly Coll. // Syntype // PARALECTOTYPUS *Gonioctena salicis* Motschulsky, 1860 des. L.N. Medvedev 2006 // *Gonioctena nivosa* (Suffrian, 1851) det. H.W. Cho.

Phytodecta nivosa var. rufula: Type depository unknown (possibly in SDEI).

Phytodecta affinis var. clythroides: Syntype 1♂ (NMPC), 17 / 894. // Tirol, Coll. Gradl // TYPUS // Phytodecta nivosus TYPE ab. clytroides Gradl n. a. 1945 Det. J. Bechyně. // Mus. Nat. Pragae Inv. 19 103.

Phytodecta affinis var. marginata: Syntypes 1♂ (NMPC), 17 / 896. // Tirol, Coll. Gradl // TYPUS // Phytodecta nivosus TYPE ab. marginatus Gradl 1945 Det. J. Bechyně. // Mus. Nat. Pragae Inv. 19 114; 1♀ (NMPC), 17 / 891. // Tirol, Coll. Gradl // COTYPE // Phytodecta nivosus COTYPE ab. marginatus Gradl 1945 Det. J. Bechyně. // Mus. Nat. Pragae Inv. 19 115; 1♀ (NMPC), 17 / 892. // Tirol, Coll. Gradl // COTYPE // Phytodecta nivosus COTYPE ab. marginatus Gradl 1945 Det. J. Bechyně. // Mus. Nat. Pragae Inv. 19 116; 1♂ (NMPC), 17 / 895. // Tirol, Coll. Gradl // COTYPE // Phytodecta nivosus COTYPE ab. marginatus Gradl 1945 Det. J. Bechyně. // Mus. Nat. Pragae Inv. 19 117; 1♀ (NMPC), 17 / 897. // Tirol, Coll. Gradl // COTYPE // Phytodecta nivosus COTYPE ab. marginatus Gradl 1945 Det. J. Bechyně. // Mus. Nat. Pragae Inv. 19 118; 1♀ (NMPC), 17 / 893. // Tirol, Coll. Gradl // COTYPE // Phytodecta nivosus COTYPE ab. marginatus Gradl 1945 Det. J. Bechyně. // Mus. Nat. Pragae Inv. 19 119; 1♀ (NMPC), 17 / 899. // Tirol, Coll. Gradl // COTYPE // Phytodecta nivosus COTYPE ab. marginatus Gradl 1945 Det. J. Bechyně. // Mus. Nat. Pragae Inv. 19 120; 1♀ (NMPC), 17 / 898. // Tirol, Coll. Gradl // COTYPE // Phytodecta nivosus COTYPE ab. marginatus Gradl 1945 Det. J. Bechyně. // Mus. Nat. Pragae Inv. 19 121.

Phytodecta affinis var. nana: Syntype 1♀ (NMPC), 17 / 884. // Tirol, Coll. Gradl // TYPUS // Phytodecta nivosus TYPE ab. nanus Gradl n. ab. 1945 Det. J. Bechyně. // Mus. Nat. Pragae Inv. 19 104.

Phytodecta affinis var. nigricollis: Syntype 1♀ (NMPC), 2 / 846 // Tirol, Coll. Gradl // TYPUS // Phytodecta nivosus Suffr. a. nigricollis Gradl TYPE 1945 Det. J. Bechyně. // Mus. Nat. Pragae Inv. 19 128.

Phytodecta affinis var. octopunctata: Syntypes 1♀ (NMPC), 17 / 262. // Tirol, Coll. Gradl // TYPUS // *Phytodecta nivosus* TYPE *8-punctatus* Gradl n. ab. 1945 Det. J. Bechyně. // Mus. Nat. Pragae Inv. 19 107; 1♂ (NMPC), 23 / 953. // Tirol, Coll. Gradl // COTYPE // *Phytodecta nivosus* Suffr. COTYPE ab. *8-punctatus* Gradl 1945 Det. J. Bechyně. // Mus. Nat. Pragae Inv. 19 108; 1♂ (NMPC), 17 / 263. // Tirol, Coll. Gradl // COTYPE // *Phytodecta nivosus* COTYPE ab. *8-punctatus* Gradl 1945 Det. J. Bechyně. // Mus. Nat. Pragae Inv. 19 109.

Phytodecta affinis var. tyrolensis: Syntype 1♀ (NMPC), 17 / 890. // Tirol, Coll. Gradl // TYPUS // Phytodecta nivosus TYPE a. tyrolensis Gradl n. ab. 1945 Det. J. Bechyně. // Mus. Nat. Pragae Inv. 19 127.

Phytodecta nivosa var. aethiops: Type depository unknown (possibly in SDEI).

Phytodecta nivosa var. apicalis: Type depository unknown (possibly in SDEI).

Phytodecta nivosa var. eppelsheimi: Syntypes 1♂ (ZMHB), Stilfser Joch...[illegible] // *stenomera* Dufour, *eppelsheimi* m. // ex. coll. J. Weise; 1♂ (ZMHB), Brenner // Strasser // ex. coll. J. Weise; 1♂ (ZMHB), Stilfser Joch. Mts. Cristallo, v. Bodemeyer // ex. coll. J. Weise; 1♀ (ZMHB), *nivosa*, *eppelsh*. // ex. coll. J. Weise; 1♀ (ZMHB), ex. coll. J. Weise.

Phytodecta nivosa var. funesta: Type probably lost.

Phytodecta nivosa var. personata: Syntypes 1♂ (ZMHB), Tirol // v. *personata* // ex. coll. J. Weise; 1♀ (ZMHB), Savoyen, manuel // ex. coll. J. Weise; 2♂♂, 1♀ (ZMHB), ex. coll. J. Weise; 1♂ (SDEI), Tirol, Reitter // 323 // v. *personata* Weise.

*Gonioctena scutellaris*: Holotype ♂ (NHRS), Porte Clarence (Alaska) // Exped. Vega. // Spec. typ. // 206 // Typus // *Gonioctena scutellaris* J. Sahlb // NHRS-JLKB 000023152 // *Gonioctena nivosa* (Suffrian, 1851) det. H.W. Cho.

Phytodecta nivosa var. ruficollis: Syntype 1♀ (ZMHB), Brenner // Strasser // var. *ruficollis* // ex. coll. J. Weise.

*Phytodecta linnaeana bergrothi*: Lectotype ♂ (ZIN), hereby designated, Fl. Jenisej // J. Sahlb. // J. Sahlberg 900. // *linnaeana bergrothi* // LECTOTYPUS *Phytodecta linnaeana bergrothi* Jacobson, 1901 des. H.W. Cho 2014 // *Gonioctena nivosa* (Suffrian, 1851) det. H.W. Cho 2014.

Phytodecta nivosa var. cedehensis: Type probably in MSNM.

Phytodecta affinis var. decaspilotus: Syntype 1♀ (NMPC), Norv. Dowre ex coll. Donckier // *Phytodecta affinis* Sch! J. Achard det in Mars // Coll. Achard Mus. Pragense // TYPUS // Phytodecta affinis TYPE ab. decaspilotus Achard 1945 Det. J. Bechyně. // Mus. Nat. Pragae Inv. 19 085 // *Gonioctena nivosa* (Suffrian, 1851) det. H.W. Cho 2014.

Phytodecta affinis var. hamatus: Syntype 1♂ (NMPC), Lapponia // J. Sahlb. // Phytodecta s. str. affinis J. Achard det. // Coll. Achard Mus. Pragense // TYPUS // Phytodecta affinis TYPE ab. hamatus Achard 1945 Det. J. Bechyně. // Mus. Nat. Pragae Inv. 19 090 // *Gonioctena nivosa* (Suffrian, 1851) det. H.W. Cho 2014.

Phytodecta nivosus var. immarginatus: Syntypes 1♂ (NMPC), Helvetia // Coll. Achard Mus. Pragense // TYPUS // Phytodecta nivosus TYPE ab. immarginatus Achard 1945 Det. J. Bechyně. // Mus. Nat. Pragae Inv. 19 123; 1♂ (NMPC), Helvetia, Reitter. // Coll. Achard Mus. Pragense // COTYPE // Phytodecta nivosus COTYPE ab. immarginatus Achard 1945 Det. J. Bechyně. // Mus. Nat. Pragae Inv. 19 124; 1♂ (NMPC), Helvetia // Coll. Achard Mus. Pragense // COTYPE // Phytodecta nivosus COTYPE ab. immarginatus Achard 1945 Det. J. Bechyně. // Mus. Nat. Pragae Inv. 19 125.

Phytodecta linnaeanus var. simplex: Type probably lost.

Phytodecta nivosa var. undulata: Syntype 1♂ (MNHN), Col du Pallet // type // v. *undulata* Pic // TYPE // Museum Paris Coll. M. Pic // SYNTYPE Phytodecta viminalis var. undulata Pic, 1924.

*Phytodecta dinah*: Holotype ♂ (NMPC), Sibérie, coll. Donckier // *Phytodecta dinah* TYPUS n. sp. ♂ 1945 Det. J. Bechyně. // TYPUS // Coll. Achard Mus. Pragense // Mus. Nat. Pragae Inv. 19 079 // Gonioctena (Gonioctena) dinah (Bechyně) Det. S. GE 2004 // *Gonioctena nivosa* (Suffrian, 1851) det. H.W. Cho 2014.

*Gonioctena arctica alberta*: Holotype (CNCI), not examined. Paratypes 1♂ (CNCI), Glacier Park Mont., 23 July 1924 // PARATYPE *Gonioctena arctica alberta* Brown, No. 6006; 1♀ (CNCI), Nordegg, Alta., 10.VI.1921, J. McDunnough // PARATYPE *Gonioctena arctica alberta* Brown, No. 6006.

Phytodecta flavicornis var. limbatipennis: Syntype 1♀ (NMPC), Schlüsseljoch [= Allemagne] // Germania Reitter // TYPUS // Phytodecta flavicornis TYPE ab. limbatipennis Ach. 1945 Det. J. Bechyně. // Coll. Achard Mus. Pragense // Mus. Nat. Pragae Inv. 18 906 // *Gonioctena flavicornis* (Suffrian, 1851) det. H.W. Cho 2013.

Phytodecta nivosa var. bicolor: Syntypes 2♀♀ (SDEI), 507. // Engadin, Strl // Phytodecta nivosa var. bicolor Heyden, 1883 // *Gonioctena flavicornis* (Suffrian, 1851) det. H.W. Cho 2014.

#### Other material.

**Norway**: 1♂, 2♀♀ (NHMB), Umg. Tromso, Norwegen; 1♂, 1♀ (NHMB), J. Schneider, Tromso // Ovoviviparous, det. H.W. Cho 2014; 1♀ (NHMB), Ivalo, Finland; 1♀ (BMNH), N Norway: Arnoy, VI.–VII.1958, P.J.M. Greenslade, B.M. 1969-168.; 1♀ (AWC), Norvegia, ad Tromso, 1898; 1♂ (NMPC), Norge, 7.13; 1♂ (NMPC), Norv. Dowre // Coll. Achard Mus. Pragense // Mus. Nat. Pragae Inv. 19 086. **Sweden**: 1♂ (NMPC), Lpl. Abisko, 21.VI.–2.VII.1948, T.Palm leg.; 1♂ (BMNH), Lapland. S. of Riksgransen, Vindskydd [= Karsatjakko], 800–900 m. VII–VIII.1957 // N. SWEDEN: B.G. Gardiner. B.M.1957-657; 1♂ (SDEI), Lappland, Kvikkjokk, 24.VI.–7.VII.1901, Thurau S. **Finland**: 1♂ (ZMHB), Halssch. Eppelsh. // ex. coll. J. Weise; 1♂ (MZHF), Fl. Nuorti, Envald; 1♀ (MZHF), Petsamo, Hellén; 1♂ (SDEI), Lac. Inari // Thuneberg // Finland; 1♀ (NMPC), Ivalo // Thuneberg // Finland // Mus. Nat. Pragae Inv. 19 088; 1♂ (NMPC), Syd-Varanger // Fennia // Krogerus // Coll. Achard Mus. Pragense // TYPUS // Phytodecta affinis TYPE ab. fennicus n. ab. 1945 Det. J. Bechyně. // Mus. Nat. Pragae Inv. 19 089; 1♀ (TLMF), Utsjoki, leg. Hellén; 1♂ (TLMF), Kilpisjärvi, leg. V. Löfgren; 1♂ (TLMF), Kevo, 23.VI.1989. **France**: 4♂♂ (MNHN), Parc National de la Vanoise, 16.VII.1897; 1♀ (NMPC), Berarde – 1925, Hautes – Alpes, Ga. ing. Jedlička; 1♂ (TLMF), Massif du Mt. Cenis, la Petit Turra, 2500m, 22.VII.2003, leg. Knapp; 1♂ (TLMF), Vanoise, Pralognan, Ref. du Grd. Bec, 2400m, 19.VII.2002, leg. Knapp; 1♂, 1♀ (TLMF), Haute Maurienne, Bonval, Sentier Balcon, 2600m, 27.VII.2003, leg. Knapp; 1♀ (TLMF), Col de la Bonette, 2500m, 26.VII.1977, leg. Kippenberg; 1♂ (TLMF), F/73 Savoie, Haute, Maurienne, Bonval, Sentier Balcon 2600 m, 27.VII.2003, leg. Knapp. **Switzerland**: 1♂, 4♀♀ (NHMB), Vals, Switzerland, VII-1925; 1♂, 1♀ (NHMB), Vals, Switzerland, 11.VII.1925; 3♂♂, 2♀♀ (NHMB), Vals, Tomül, Switzerland, 11.VII.1908; 3♂♂, 1♀ (NHMB), Switzerland, Val Tasna, Engadin, 12.VII.1949, W. Schlier; 3♂♂, 1♀ (NHMB), GR Sur, Alp Flix CH, Val Savriez, Plan Bel, 2400 m, 771.8/154.5, 20.VI.2002, leg. W. Marggi; 1♂ (ABC), Mt. Rosa. W. Bohmlander // Helvetia mer.; 5♂♂ (BMNH), Arolla, Switz. G.C.C. // G.C. Champion Coll. B.M. 1927-409; 1♂ (AWC), Helvetia (Wallis), mons Eggis-Horn, leg. B. Malkin; 1♂ (NMPC), Val. Piora (Switzerland), E. 6. 08; 1♀ (TLMF), Churfirsten, Brisi, 2260m, 27.VI.2004, leg. Kapp; 1♀ (TLMF), Unterwalden: Susten-Pass, 2200–2400m, 23.VII.1991, leg. Hiermeier; 7♂♂, 4♀♀ (TLMF), Umbrail-Pass, 2500–2700m, 4.IX.1974, leg. Kippenberg; 2♂♂, 1♀ (TLMF), Umbrail-Pass, 2300–2500m, 16.VIII.1975, leg. Kippenberg; 1♀ (TLMF), Stilfser Joch, 2700m, 6.IX.1986, leg. Kippenberg; 1♂ (TLMF), Albula-Pass, 2000–2200m, 6.VI.1993, leg. Kippenberg; 1♂ (TLMF), Greina-Ebene, 2300m, 5.V.1988, leg. W. Marggi; 1♂ (TLMF), Greina-Gebiet, 2500m, VII.1988, leg. W. Marggi; 1♂ (TLMF), Greina-Süd, Alp Motterascio 2200m, VII.1988, leg. W. Marggi; 1♂ (TLMF), Oberalp-Pass, 2100–2200m, 23.VII.1991, leg. Hiermeier; 1♀ (TLMF), Furka-Pass, 2450m, 30.VII.1982, leg. Kippenberg; 1♂, 1♀ (TLMF), Vispertinem, Gebidem, Nanztal, 2400m, 23.VI.2002, leg. W. Marggi; 1♀ (TLMF), Zinal, Come de Borebois, 2800m, 25.VI.2002, leg. Gollkowski; 1♂, 2♀♀ (TLMF), Grimsel-Pass, 2180m, 26.IX.1990, leg. I. Wolf. **Austria**: 1♂ (NHMB), Austria, Tilisuna See, Montafon, 18–2200 m, leg. Dr. Mandl, VII.1954; 1♂, 1♀ (ABC), Bachlenke Troyer Tal hochalpin // Osttirol Holdhaus; 1♀ (SDEI), Austria: S Ferleiten (Hohe Tauern), 47°07’27’’N, 12°49’17’’E, 18.VII.1999, 2300 m, leg. C. Lange & J. Ziegler; 2♂♂ (SDEI), 1♂ (TLMF), Austria: Salzb., Hohe Tauern, Fusch, 2300 m, 6.VII.1993, leg. Zerche; 1♀ (NMPC), Tirol // Collectio A. Fleischer // TYPUS // Phytodecta nivosus TYPE ab. latefasciatus n. ab. 1945 Det. J. Bechyně. // Mus. Nat. Pragae Inv. 19 136; 1♀ (NMPC), Rhaetia // Collectio A. Fleischer // TYPUS // Phytodecta nivosus TYPE ab. limitata n. ab. 1945 Det. J. Bechyně. // Mus. Nat. Pragae Inv. 19 126; 1♀ (NMPC), 17 / 889. // Tirol, coll. Gradl // *Phytodecta nivosus* Suffr. 1945 Det. J. Bechyně. // Mus. Nat. Pragae Inv. 19 091; 1♀ (NMPC), 17 / 885. // Tirol, coll. Gradl // Phytodecta nivosus TYPE ab. excisus n. ab. 1945 Det. J. Bechyně. // Mus. Nat. Pragae Inv. 19; 7♂♂, 5♀♀ (ZMUC), Österreich, Triol, Ötztaler, Alpen: Vent 7 km S, Umg. Martin-Busch-Hutte (2700 m, feuchtes Schotterfeld, saß auf Stein), 01.VII.2008, leg. D. Luckow; 1♀ (TLMF), Ulmer Hütte, 30.VI.1928, leg Ratter; 1♂ (TLMF), Kühtai, Feldringer Alm, 25.VI.2006, leg. M. Egger; 1♂ (TLMF), Ötztal, Chemnitzer Hütte, 2600m, 2.IX.1951; 1♂, 1♀ (TLMF), Ötztal, Kreuzspitze, 3000m, 5.VIII.1948; 1♂ (TLMF), Ötztal, Obergurgl, Gaisbergtal, 2200–2400m, 17.IX.1997, leg. Kippenberg; 1♀ (TLMF), Nößlachjoch, 2100m, 28.IX.1975, leg. K. Burmann; 1♂ (TLMF), Hohe Tauern, Innergschlöß, Prager Hütte, 2300–2400m, 5.IX.1983, leg. Kippenberg; 2♀♀ (TLMF), Hohe Tauern, Kals, Ködnitztal, Fanatscharte, 2600–2700m, 3.IX.1985, leg. Kippenberg; 1♀ (TLMF), Hohe Tauern, Matrei, Tauernhaus, 11.VII.1991, leg. M. Egger; 1♂ (TLMF), Schladminger Tauern, Steirische Kalkspitze, 2200m, 15.VIII.1986, leg. Kippenberg; 1♂ (TLMF), Hohe Tauern, Heiligenblut, Pasterzenhaus, 2200m, 10.VII.1993, leg. Zerche. **Italy**: 2♂♂, 2♀♀ (JBC), Italy, Val d’Ayas (Aosta), 12.VII.1978, S Zoia; 1♂ (ABC), Vinschgau. Ti.G.Kuchta // Italien S.-Tirol; 1♂ (TLMF), I-Cuneo, Alpi Cozie, Colle dell’Agnello, leg. Kahlen // S-Seite 2700 m, 20.VI.2000, Schneeboeen, Salix herbacea-Rasen; 1♂ (SDEI), Lombardia, Val Brembana, Lago Colombo, L. Ceresa // coll. K. H. Mohr, DEI Eberswalde; 1♂ (SDEI), Fiery d’Ayas, Val d’Aosta, VII.1910, A. Dodera // O. Leonhard; 1♂ (SDEI), Italy, Sudtirol, 5.VII.1928, Linke leg.; 1♀ (DBET), Stelvio; 1♀ (NMPC), Alpy Penninske. Italia; 1♂ (NMPC), Stelvio Ti, 15.7.05 // TYPUS // Phytodecta nivosus TYPE ab. marginipennis n. ab. 1945 Det. J. Bechyně. // Mus. Nat. Pragae Inv. 19 134; 5♂♂ (ZMUC), Italien, Lombardia: Stilfser Joch (P. so di Stelvio) 1,5 km NW (Ri. Umbrailpass) (2600 m, Mattenbereich, viel Schnee, unter einem Stein) leg. D. Luckow, 12.VI.2008.102; 2♀♀ (TLMF), Umgebung Brenner, Schlüsseljoch, 25.VI.1964, leg. Kippenberg; 1♀ (TLMF), Cogne, Valnontey, Rif. V. Sella, Lago del Loson, 2660m, 8.VII.2002, leg. Kopetz; Valle d’Aosta; 1♀ (TLMF), Piccolo S. Bernardo, 2100m, 29.VI.1976, leg. Krätschmer; 1♀ (TLMF), Picc. S. Bernardo, 2100m, 13.VI.1981, leg. Kippenberg; 4♂♂ (TLMF), Alpie Cozie, Colle dell’ Agnello, 2700m, leg. Kahlen, Salix herbacea-Rasen; 1♀ (TLMF), Colle del Mulo, 2400m, VII.1968. **Slovenia**: 1♂ (AWC), Slovenia, Alpi Julian. Cervinia, 7.VII.1970. **Russia**: 1♀ (NHMB), Tschita, Transbaikal, Mandi // Ovoviviparous, det. H.W. Cho 2014; 1♂ (NHMB), Vladivostok, Russia, 1933, N. Filippov; 2♂♂ (NHMB), Russia, Taymyrsky Dolgano-Nenetsky District, Maimecha River, 7.VII.1971; 2♂♂ (JBC), Russia, Altai rep., Kalguty & Ak-Alacha junction Ukok plateau, 2150–2500 m, 49°23’N, 87°38–40’E, 8–12.VII.2009, L. Čížek leg.; 5♂♂ (ABC), Russia, Tuva, S. Slopes of E. Tanu-Ola Mts., envir. Soglyi vill., 1800–2700 m, 14.V.–24.VI.2002, Vashchenko leg.; 1♂ (ABC), Subpolar Ural Mts., Northern Maldy ridge, 1–18.VII.2000, A.A. Medvedev; 1♂ (ABC), Transbaikal Krai, Kodarsky ridge, 40 km NW from Chara Vill., 16–25.VII.1996, A.E. Brinev leg.; 1♂ (ABC), Murmansk reg., Tuloma river and Not-lake, 4–6.VIII.1906, Soldatov leg.; 3♂♂, 1♀ (HCC), Russia, S Siberia, SW Tuva reg., W Tannu Ola Mt. mg., Sogly v., 2000 m, 5.VII.2003, S. Vastchenko leg.; 1♀ (HCC), Yakutia, Chandyga distr., 7/93; 1♂ (FKC), Russia, S Siberia, SW Tuva reg., S Tannu-Ola Mts. 1800 m, 14.V.2002; 1♂ (FKC), USSR-Tungur, Gorno altaysk, 5.VII.1990, V. Lenserk lgt.; 1♂ (LMC), Kamchatka, 22.VI.1958, L. Ivliev coll.; 1♂ (SDEI), Altai // COTYPUS // Coll. Kraatz // Phytodecta affinis v. pernigra m.; 1♂ (AWC), Russia (Sib. Occ.), Kuznetzky Alatau, Malyj Zub, 31.VII.1996, leg. J. Mikhailov; 1♂ (AWC), Russia, W. Siberia, Kuznetsky Alatau mts., Malyj Zub mt. On *Salix*, 31.VII–1.VIII.1996, Yu. Mikhailov leg.; 13♂♂, 14♀♀ (TLMF), Yakutien, Chandyga, VII.1993, leg. Naglis; 1♀ (TLMF), Amur region, Selemdgin distr., Tamsche, 7.VI.2005, leg. Ryvkin & Veselova; 2♂♂, 1♀ (TLMF), Chabarowsk region, Ochotsk, Ulia river, 29.VII.–6.VIII.1985, leg. Ryvkin & Veselova; 1♂ (TLMF), SW Tuva, W Tanu-Ola Mts., near Solglyi vill., 2000–2800m, 13.V.–1.VI.2003, leg. Vashchenko; 1♂ (TLMF), Tuva, Chandagayt, 10.VII.1971; 2♂♂ (TLMF), Tuva, 80km S Taly, 23–26.VI.1972, leg. Korotyaev; 1♂ (ZIN), Fl. Jenisej // J. Sahlb. // 1274 // J. Sahlberg 900. // linnaeana bergrothi v. correspondens // *Gonioctena nivosa* (Suffrian, 1851) det. H.W. Cho 2014; 1♂ (ZIN), Russia, Khabarovsk Krai, Shantar Islands, 22.VII.1911, V. Soldatov leg.; 2♂♂ (ZIN), Russia, border of Irkutsk Oblast and Republic of Buryatia, approx. 51–52°N, 101–102°E, between the rivers Kitoy, Bogdashka and Belaya, 20.V.1973, Gartung leg.; 1♂ (ZIN), Russia, Murmansk Oblast, Kildin Island, 29.VII.1900, Il’in leg.; 1♂ (ZIN), Russia, Republic of Tuva, 80 km N Teeli, 25.VI.1972, B. Korotyaev leg.; 2♂♂ (ZIN), Ochotsk // F. Sahlb // J. Sahlberg 900; 1♂ (ZIN), Russia: Altai Kuray Mt. R., NE Aktash, upp. Tarlyamry R., 50°19’10’’N, 87°45’14’’E, 2100–2300 m, 30.VI.2005, B. Kataev leg.; 1♂ (ZIN), Russia, Krasnoyarsk Krai, Nizhnyaya Tunguska River, upper than Vivi River mouth, 28.VII.1873, Chekanovsky leg.; 1♂ (ZIN), Russia, Amur Oblast, Zeyskiy District, upper of Erakingra River, on Tukuringra Mountains, 19.VI.1957, Zinoviev leg.; 1♂ (ZIN), Russia, Amur Oblast, Zeyskiy District or Magdagachinsky District, Ulunga River, 25.V.1910, Mishin leg.; 2♂♂ (ZIN), Russia, Murmansk Oblast, Kolsky District, Notozero Lake and Tuloma River, 4–6.VIII.1906, Soldatov leg.; 1♂ (ZIN), Russia, Murmansk Oblast, Kolsky District, Nota [= Noti] River, 28.VII.1906, Soldatov leg.; 1♂ (ZIN), Russia, Krasnoyarsk Krai, Evenkiysky District, Podkamennaya Tunguska River, Big (7-verst) rapids, approx. 61N 94E, 20.VI.1928, Valdaev leg.; 2♂♂ (ZIN), Russia, Krasnoyarsk Krai, Evenkiysky District, Podkamennaya Tunguska River, Muchnoi rapids, 61.81N, 94.43E, 25.VI.1928, Valdaev leg.; 2♂♂ (ZIN), Russia, Komi Republic, Troitsko-Pechorsky District, Aranets River, 25–26.VI.1905, Zhuravskiy leg.; 1♂ (ZIN), Russia, Komi Republic, Troitsko-Pechorsky District, Bolshaya Synya River, Mount. “Voi”, Sablya, “Izb.”, 16.VI.1908, Zhuravsky leg.; 1♂ (ZIN), Russia, Sakha Republic, Verkhoyansky District, tundra along river Dogdo, Yana River basin, 16–18.VI.1901, Hertz leg.; 1♂ (ZIN), Russia, Sakha Republic, 17.VII.1925, L. Bianki leg.; 3♂♂, 1♀ (ZIN), Russia, Tyumen Oblast, Yamalo-Nenets Autonomous Okrug, Priuralsky District, Sob’ River basin, Obdorskiy Krai, 15.VII.1925, Fridolin leg.; 1♂ (ZIN), Russia, Tyumen Oblast, Yamalo-Nenets Autonomous Okrug, Shuryshkarsky District, between Varchaty Lake and Maly Ural Mountains, Obdorskiy Krai, 4.IX.1925, Fridolin leg.; 1♂ (ZIN), Russia, Sakhalin Oblast, Sakhalin Island, Holmsky pass (approx. 47°N, 142°E), 1.VII.1982, Smirnov leg. **Kazakhstan**: 1♂ (JBC), Kazakhstan, East, 2300–2500 m, 49°30’N, 86°22’E, 13–14.VI.2006; 1♂ (TLMF), Ussuria, ad chasan, 42°25’N, 130°45’E, 1–8.VII.2000, Melnik; 1♂ (TLMF), RU: Sibirien, E-Sayan 54 km west, Mondy, 8.VII.2012, 1900 m, St. FloBmann leg.; 1♂, 1♀ (TLMF), Kazakhstan, Altaj, Sarym-Sakty, 2500 m, 11.VI.1999, leg. Vashchenko; 2♂♂, 2♀♀ (TLMF), 100km SSE Ust-Kamenogorsk, Panteleymonovka vill., 16–21.VI.1993, leg. A. Napolov. **Mongolia**: 1♂ (ZIN), Mongolia, NE, Khentii Mountains, Rivers Manza and Sharotay, 19.VII.1897, Klementz leg. **Canada**: 1♂ (ABC), Aklavik, N.W.T., 24.VI.1956, E.F. Cashman leg.; 2♂♂ (CNCI), ALB., Banff Nat. Pk. Sunwapta Pass, 9.VII.1955, W.J. Brown; 6♂♂, 5♀♀ (CNCI), Reindeer Depot, Mackenzie Delta, 28.VI.1948, W.J. Brown; 20♂♂, 5♀♀ (CNCI), Aklavik, N.W.T., 16.VI.1956, E.F. Cashman; 2♂♂, 1♀ (CNCI), Saw Mill Bay, N.W.T., 16.VI.1948, D.F. Hardwick // on willow; 11♂♂, 7♀♀ (CNCI), Churchill. Man., 3.VII.1937, W.J. Brown // On *Salix*; 2♀♀ (CNCI), Toad River, B.C. Mi440 Alaska Hwy, 19.VI.1959, 4500’ R.E. Leech; 6♂♂, 1♀ (CNCI), Kluane, Y.T., 28.VII.1948, Mason & Hughes; 1♂, 3♀♀ (CNCI), North Richardson Mts. Yukon, VII.1982, On Salix m. polaris, D.M. Wood; 4♂♂, 2♀♀ (CNCI), Y.T., British Mts. June Cr. 600 m, 69°14’N, 140°08’W, J.M. Campbell; 1♂, 1♀ (CNCI), Banff Natl. Pk., Alta, 9.VII.1955, W.J. Brown // On *Salix*. **United States**: 9♂♂, 11♀♀ (CNCI), Moose Pass, Kenai Pen., 30.VI.1951, W.J. Brown; 3♂♂, 6♀♀ (CNCI), Paxon Lodge, Gulkana, Alaska, 4.VIII.1951, W.R.M. Mason; 2♂♂, 3♀♀ (CNCI), Summit Lake, B.C. Mi392 Alaska Hwy, 26–27.VI.1959, 4500’ R.E. Leech; 1♂ (CNCI), Niwot Ridge, COLO. nr. Ward, 11,500’, 4.VI.1961, W.R.M. Mason; 1♂ (TLMF), USA - Alaska, Fairbanks, 3.VIII.2009, K. Renner // Murphy Dome, 870 m, Shrubs; 1♂ (ZMUC), Colo. Morr. // Col. H. H. Meeske // Zool. Museum DK Copenhagen. **Uncertain localities**: 1♀ (NMPC), Alpes // Coll. Achard Mus. Pragense // TYPUS // Phytodecta nivosus TYPE a. vicinus n. ab. 1945 Det. J. Bechyně. // Mus. Nat Pragae lnv. 18 113; 1♂ (NMPC), Alpes // nivosus v. trinotatus m. // Coll. Achard Mus. Pragense // TYPUS // Phytodecta nivosus TYPE ab. trinotatus [Achard i. l.] n. ab. 1945 Det. J. Bechyně. // Mus. Nat. Pragae Inv. 19 105; 1♂ (NMPC), Alpes // Coll. Achard Mus. Pragense // TYPUS // Phytodecta nivosus TYPE ab. hexangularis n. ab. 1945 Det. J. Bechyně. // Mus. Nat. Pragae Inv. 19 106; 1♂ (NMPC), N America // Coll. Achard Mus. Pragense // Mus. Nat. Pragae Inv. 19 081.

#### Diagnosis.

*Gonioctena nivosa* differs in having small body size, long antennae, first tarsomere of all legs in male much strongly swollen, aedeagus moderately narrowed apically in dorsal view and strongly curved in lateral view.

#### Redescription.

Measurements in mm (n = 10): length of body: 4.05–6.00 (mean 5.19); width of body: 2.25–3.60 (mean 3.03); height of body: 1.50–2.50 (mean 2.10); width of head: 1.30–1.65 (mean 1.51); interocular distance: 0.90–1.17 (mean 1.06); width of apex of pronotum: 1.55–1.87 (mean 1.72); width of base of pronotum: 1.92–2.85 (mean 2.51); maximum width of pronotum: 1.97–2.85 (mean 2.49); length of pronotum along midline: 1.02–1.47 (mean 1.29); length of elytra along suture: 2.90–4.40 (mean 3.77).

Body oblong oval and moderately convex (Fig. 7). Coloration extremely variable. Head black. Mandibles black, with reddish brown band near apex. Maxillary palps reddish brown or dark brown, with apical palpomere black. Antennomeres 1–5 yellowish brown, partially darkened, 6–7 darkened, 8–11 dark brown or blackish brown. Pronotum entirely black or reddish brown with a large black marking, rarely entirely reddish brown (Figs 31–32). Scutellum black, rarely entirely reddish brown. Elytra entirely black or reddish brown, with or without 4–5 pairs of black spots. Venter black, with hypomera reddish brown or black and apical margin of last abdominal ventrite reddish brown. Legs black, with tibiae reddish brown except base and inner margin and tarsi blackish brown or reddish brown, sometimes legs entirely black to dark brown.

**Figures 29–32.**
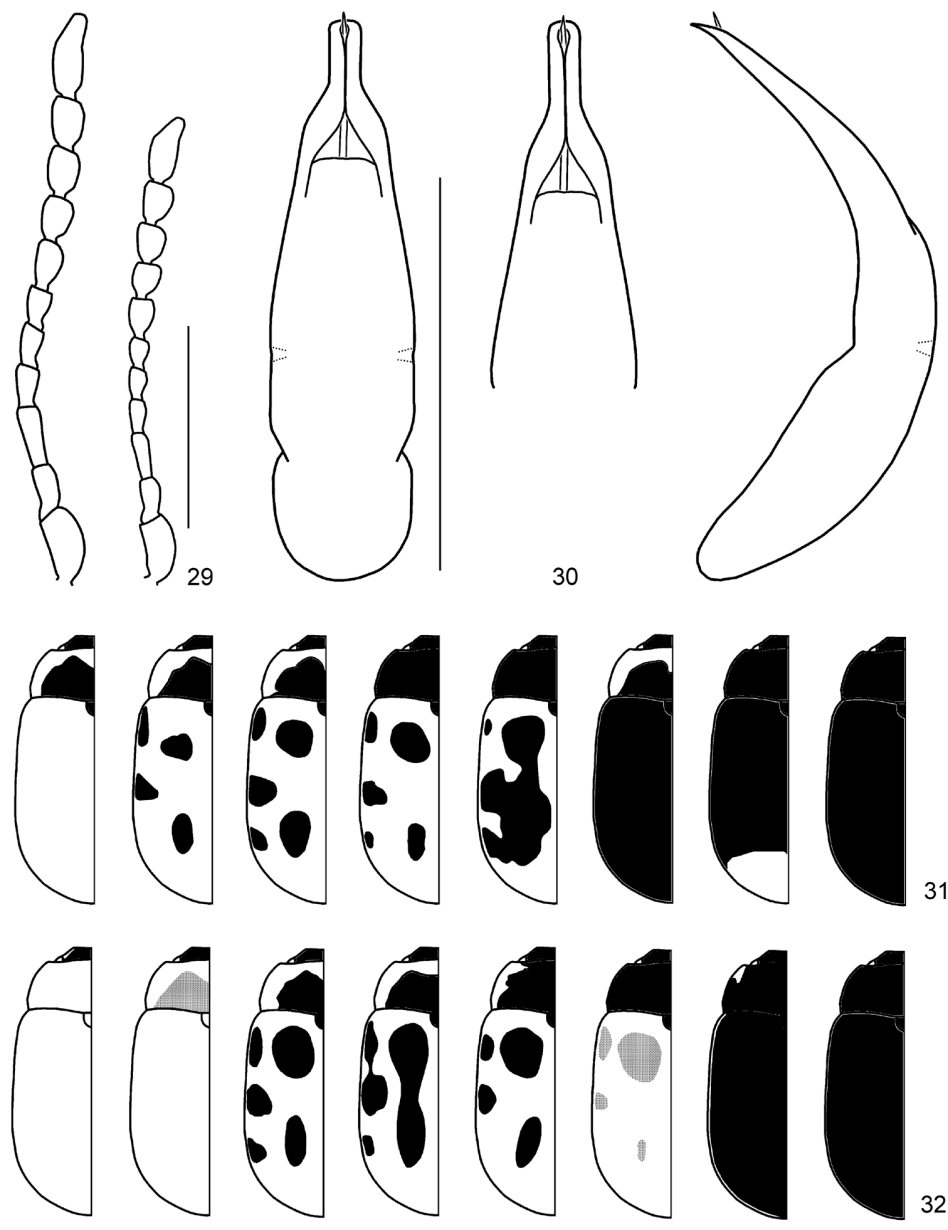
*Gonioctena nivosa*. **29** Antenna (♂, ♀) **30** Aedeagus (Hohe Tauern, Austria) **31** Color variation (Palaearctic region) **32** Color variation (Nearctic region). Scale bars = 1.0 mm.

*Head*. Vertex weakly convex, covered with dense punctures. Frontal suture V-shaped, coronal suture weak or absent. Frons flat, strongly depressed at anterior margin, covered with dense punctures. Clypeus narrow and trapezoidal. Anterior margin of labrum distinctly concave. Mandibles with 2 sharp apical teeth and a deep excavation for apical maxillary palpomere at outer side. Maxillary palps 4-segmented, with apical palpomere distinctly widened, truncate apically in male; slightly widened in female. Antennae in male longer than half length of body; antennomere 1 robust; antennomere 2 shorter than 3; antennomere 3 longer than 4; antennomeres 7–11 each distinctly longer than wide; antennomere 11 longest, about 2.68 times as long as wide (Fig. 29). Antennae in female reaching elytral humeri; antennomere 11 about 2.31 times as long as wide.

*Pronotum*. Lateral sides widest near base, roundly moderately narrowed anteriorly, anterior angles strongly produced (Fig. 8). Anterior and lateral margins bordered, lateral margins not or hardly visible in dorsal view. Trichobothria present on posterior angles. Disc covered with rather dense punctures; lateral sides covered with much coarser and denser punctures, becoming larger toward base, partially confluent near basal margin; interspaces covered with fine and sparse punctures. Scutellum slightly wider than long, narrowed posteriorly.

*Elytra*. Lateral sides moderately widened posteriorly, widest beyond middle, thence roundly narrowed posteriorly. Humeral calli well developed. Disc covered with 11 regular rows of large punctures, including a short scutellar row; sometimes punctures rather irregular between 6th and 8th striae in apical half; interspaces shagreened, covered with fine and sparse punctures. Epipleura wholly visible in lateral view. Hind wings well developed.

*Venter*. Hypomera weakly rugose, with a few punctures near anterolateral corners of prosternum. Prosternum covered with coarse and dense punctures bearing long setae; prosternal process enlarged apically, bordered laterally, with sparse punctures. Metasternum covered with small and sparse punctures in median region, large and dense punctures in lateral region. Abdominal ventrites covered with moderately dense punctures bearing short setae.

*Legs*. Moderately robust. Tibiae widened apically, with a tooth-like projection. Fore legs with tarsomere 1 strongly enlarged, distinctly wider than 3 in male; very slightly narrower than 3 in female. Tarsal claws appendiculate.

*Genitalia*. Aedeagus moderately narrowed apically, with apical process rather short in dorsal view; strongly curved, with apical process pointed and slightly bent downward at apex in lateral view (Fig. 30). Spermatheca absent.

#### Distribution.

Transholarctic species: Austria, Finland, France, Germany, Italy, Kazakhstan, Liechtenstein, Norway, Slovenia, Spain, Sweden, Switzerland, Mongolia, Russia (North European Territory, West & East Siberia, Far East, Sakhalin), Canada (Alberta, British Columbia, Manitoba, Northwest Territories, Yukon), United States (Alaska, Montana).

#### Host plant.

Salicaceae: *Salix* spp. (Mannerheim 1853, Motschulsky 1860, Stål 1865, Brown 1942, Wilcox 1972, L. N. Medvedev and Zaytsev 1978, L. N. Medvedev and Roginskaya 1988, L. N. Medvedev 1992, L. N. Medvedev and Dubeshko 1992, Steinhausen 1994, Bieńkowski 2004, Zaytsev and L. N. Medvedev 2009); *Salix retusa* (Reitter 1913, Cantonnet 1968, Kippenberg 1994); *Salix retusa*, *Salix herbacea* (Daccordi et al. 1991); *Salix* spp., *Salix bebbiana* (Clark et al. 2004). Rosaceae: *Spiraea* spp. (L. N. Medvedev and Roginskaya 1988, L. N. Medvedev 1992).

#### Remarks.

**Figures 33–54.**
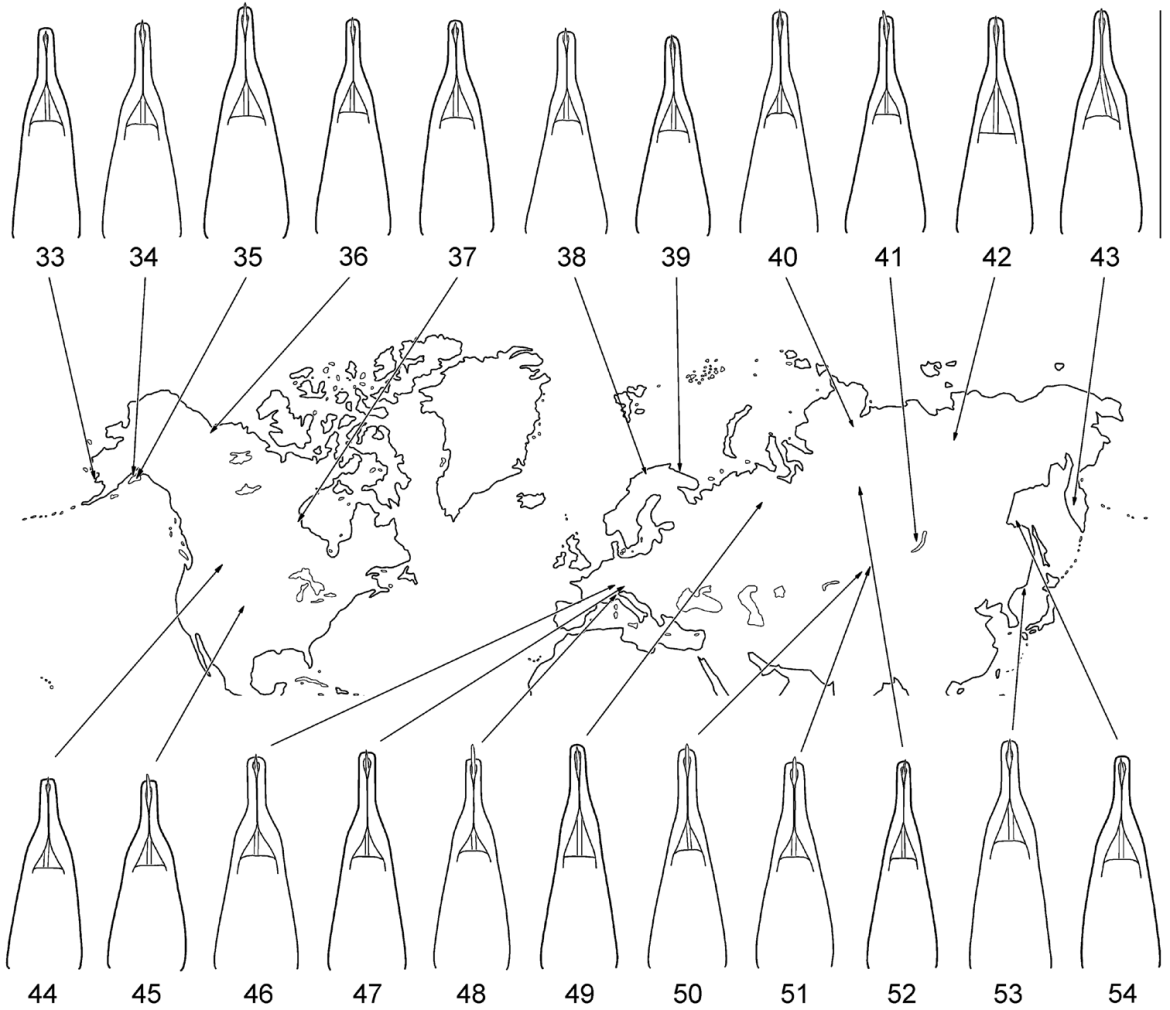
Geographic variation in male genitalia of *Gonioctena nivosa*. **33** Porte Clarence, Alaska **34** Kenai, Alaska **35** Summit Lake, Alaska **36** Aklavik, Canada **37** Churchill, Canada **38** Tromso, Norway **39** Kildin Island, Russia **40** Maimecha River, Russia **41** Baikal area, Russia **42** Verkhoyansky, Russia **43** Kamchatka, Russia **44** Glacier Park, USA **45** Niwot Ridge, USA **46** Hohe Tauern, Austria **47** Karnten, Austria **48** Lombardia Val Brembana, Italy **49** Troitsko-Pechorsky, Russia **50** E Kazakhstan **51** Altai, Russia **52** Podkamennaya Tunguska, Russia **53** Vladivostok, Russia **54** Shantar Islands, Russia. Scale bar = 1.0 mm.

The taxonomic status of *Gonioctena nivosa*, and its relationships to *Gonioctena affinis* and *Gonioctena arctica* has been disputed for a long time. Kraatz (1879a) treated *Gonioctena arctica* as a synonym of *Gonioctena nivosa*. Székessy (1934) synonymized *Gonioctena arctica* and *Gonioctena nivosa* with *Gonioctena affinis*, while Bechyně (1948) established them as distinct species. Mohr (1966) regarded *Gonioctena affinis* and *Gonioctena nivosa* as phylogenetically young group that are almost identical. Wilcox (1972) treated *Gonioctena arctica* as a subspecies of *Gonioctena nivosa*. However, *Chrysomela affinis* Gyllenhal, 1808 is a junior homonym of *Chrysomela affinis* Fabricius, 1787, therefore Silfverberg (1977) proposed the name *Gonioctena decaspilota* as the oldest available name. He again treated *Gonioctena decaspilota* and *Gonioctena nivosa* as distinct species. All these taxa have been confused until now by many authors (see list above). After examining all type specimens of the discussed taxa and many other specimens from the Holarctic region we conclude that all these taxa are conspecific. The shape of body and aedeagi from Europe, Siberia, Far East and North America are identical, although aedeagi and color patterns slightly vary even within the same population (Figs 31–54). Three type localities of *Gonioctena nivosa* are given in the original description: Kärnten, Berner Alpen and Switzerland. Due to the designation of lectotype, the restricted type locality becomes “Kärnten [= Carinthia in Austria]” (ICZN 1999: Recommendation 74E). Six paralectotypes of *Chrysomela affinis* Gyllenhal, 1808 belong to *Gonioctena linnaeana* (Schrank, 1781).

We examined the type of *Phytodecta linnaeana bergrothi* and found it is conspecific with *Gonioctena nivosa*. *Phytodecta linnaeana bergrothi* has been misidentified since its original description and is here synonymized with *Gonioctena nivosa*. The name Phytodecta linnaeanus bergrothi var. simplex published by Jacobson (1901b) is infrasubspecific. It is available from Achard (1924) who first used it for a variety of species, Phytodecta linnaeanus var. simplex (ICZN 1999: Article 45.5.1). However, this name and its incorrectly proposed replacement name Phytodecta linnaeanus var. mutatus are removed from synonymy with *Gonioctena linnaeana* and are synonymized with *Gonioctena nivosa* based on the original description. Phytodecta nivosa var. cedehensis is for the Alpine specimen having black elytra with a large yellow marking at tip and is synonymized with *Gonioctena nivosa*.

We also examined the types of Phytodecta nivosa var. bicolor Heyden, 1883 and Phytodecta flavicornis var. limbatipennis Achard, 1924 and found that they are conspecific with *Gonioctena flavicornis* (Suffrian, 1851). Therefore, they are removed from synonymy with *Gonioctena nivosa* and are synonymized with *Gonioctena flavicornis*.

Several larvae were dissected from the female specimens collected in Norway and Transbaikalia, therefore this species is ovoviviparous (Fig. 67). The previous record of the occurrence of ovoviviparity by Notman (1921) is based on misidentified *Gonioctena notmani* (Schaeffer, 1924).

### Gonioctena (Gonioctena) norvegica

Taxon classificationAnimaliaColeopteraChrysomelidae

(Strand, 1936)

Figs 9–10 , 55–61 , 62

Phytodecta norvegicus Strand, 1936: 104 (type locality: Målselv, Rundhaugen, Nordmo); Palmén 1946: 230.
Gonioctena norvegicus : L. N. Medvedev and Korotyaev 1980: 81 (as synonym of Gonioctena affinis).
Gonioctena norvegica : Silfverberg 1992: 69, 1994b: 32, 2004: 82.
Gonioctena (Gonioctena) norvegica : Bieńkowski 2004: 67; Kippenberg 2010: 434.
Phytodecta charitonowi Palmén, 1946: 231 (type locality: Siberia); Kippenberg 2010: 434 (as synonym of Gonioctena norvegica).
Gonioctena janovskii L. N. Medvedev, 1976: 234 (type locality: Mongolia, Central Aimak, Tereldzhin gol forestry); L. N. Medvedev and Voronova 1976: 229; Zaytsev and L. N. Medvedev 1977: 368 (larva); L. N. Medvedev and Zaytsev 1980: 106 (larva); L. N. Medvedev and Roginskaya 1988: 101 (host plant); Dubeshko and L. N. Medvedev 1989: 133 (biology). **syn. n.**
Gonioctena (Gonioctena) janovskii : L. N. Medvedev and Zaytsev 1978: 119 (larva); L. N. Medvedev 1982: 91, 179, 252 (incl. larva); Lopatin et al. 2004: 122; Kippenberg 2010: 433; Warchałowski 2010: 558.

#### Type material.

*Phytodecta norvegicus*: Syntypes 2♂♂, 2♀♀ (NMPC), Rundhaug M. elv, A. Strand; 1♂ (NHRS), Rundhaug M. elv, A. Strand // Paratypus // *Phytodecta norvegicus* A. Strand // NHRS-JLKB 000023154.

*Phytodecta charitonowi*: Holotype probably in MZHF.

*Gonioctena janovskii*: Holotype ♂ (LMC), Holotypus // 29.VI.1971, Mongolian People’s Republic, Central Aimak, Tereldzhin gol forestry, on *Salix* leaves, V. Yanovsky leg. Paratypes: 1♂ (LMC), same data as holotype; 1♂, 1♀ (LMC), same data as holotype except 23.VI.1971.

#### Other material.

**Finland**: 2♂♂, 2♀♀ (ZMUC), Fennia, Ob Rovaniemi, Piaa, 21.6.1915, Hakan Lindberg; 2♂♂ (SDEI), Lapponia, Leonhard leg. **Sweden**: 1♂ (ZMUC), Nb. Storsien, 22.6.1981, G. Gillerfors // *norvegicus* // Ex coll. Viggo Mahler. **Russia**: 1♂, 1♀ (ABC), Altai Mts., environs of Bayas lake, 51°17’N, 87°56’E, 1700 m, 14.VIII.1993, M. Savitsky leg.; 1♂ (ABC), Komi Republic, Intinsky Distr., Paga-ty lake, 25.VI.2007, A.A. Kolesnikova leg.; 1♂ (AWC), RUSSIA, SE Tuva, Khorummnug-Taiga Mts., Ailyg-Kai River Valley, subalpine, on *Salix*, 15.VI.1999, Yu. Mikhailov leg. // *Gonioctena janovskii* L. Medvedev, M. Bergeal det. 2000; 1♂ (ZIN), Russia, Sverdlovsk Oblast or Tyumen Oblast, Manya River basin, forest Urals, 16–19.VI.1927, Lyapin and Flerov leg.; 1♂ (TLMF), Russia, E-Sayan, Sibir, 54 km w Mondy 1900m, 13.VII.2012, leg. S. Floßmann. **Mongolia**: 3♂♂, 2♀♀ (NMPC), Mong. bor. BOGDO-UL, 11.VIII.66, Dlabola, in litt. loc. 39.

#### Diagnosis.

*Gonioctena norvegica* differs in having antennae much shorter than half length of body in male, not reaching elytral humeri in female, aedeagus rather thick in dorsal view and strongly curved in lateral view.

#### Redescription.

Measurements in mm (n = 5): length of body: 4.30–5.50 (mean 4.98); width of body: 2.35–3.40 (mean 3.00); height of body: 1.60–2.30 (mean 1.98); width of head: 1.30–1.50 (mean 1.42); interocular distance: 0.95–1.10 (mean 1.03); width of apex of pronotum: 1.47–1.72 (mean 1.62); width of base of pronotum: 2.07–2.78 (mean 2.46); maximum width of pronotum: 2.10–2.80 (mean 2.48); length of pronotum along midline: 1.15–1.35 (mean 1.24); length of elytra along suture: 2.95–4.20 (mean 3.65).

Body oblong oval and moderately convex (Fig. 9). Head black. Mandibles black, with dark reddish brown band near apex. Maxillary palps blackish brown, with apical palpomere black. Antennae yellowish brown or reddish brown, generally with last 4–6 antennomeres darkened. Pronotum reddish brown, with small or large black markings (Fig. 61). Scutellum black. Elytra reddish brown, with 4–5 pairs of black spots. Venter black, with hypomera and apical margin of last abdominal ventrite reddish brown. Legs black, with tibiae reddish brown except base and inner margin and tarsi dark brown or reddish brown.

**Figures 55–61.**
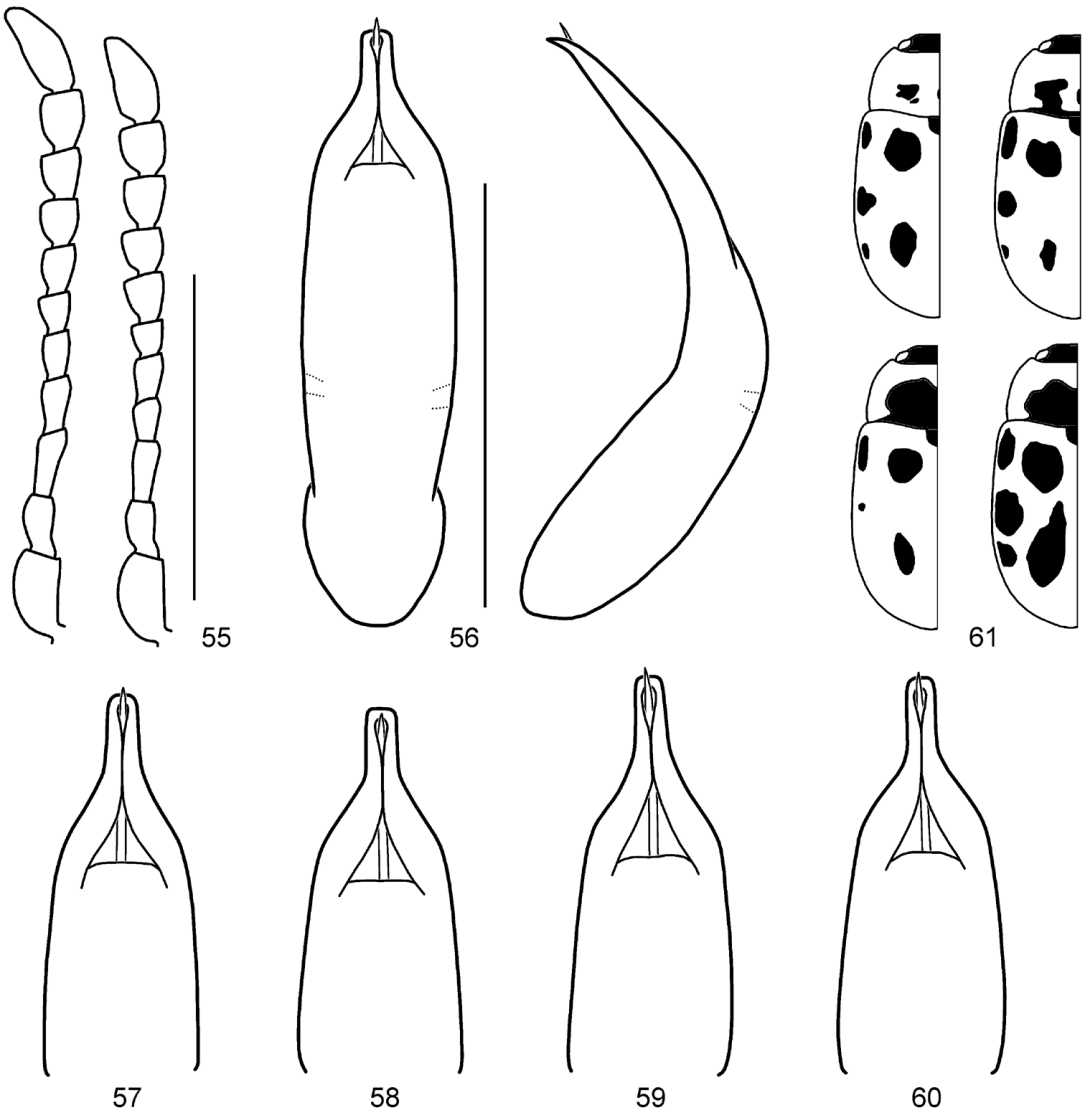
*Gonioctena norvegica*. **55** Antenna (♂, ♀) **56–57** Aedeagus (Rundhaug, Norway) **58** Aedeagus (Bogd Uul, Mongolia) **59** Aedeagus (Manya River, Russia) **60** Aedeagus (Intinsky, Komi Rupublic, Russia) **61** Color variation. Scale bars = 1.0 mm.

*Head*. Vertex weakly convex, covered with moderately dense punctures. Frontal suture V-shaped, coronal suture weak or absent. Frons flat, strongly depressed at anterior margin, covered with dense punctures. Clypeus narrow and trapezoidal. Anterior margin of labrum distinctly concave. Mandibles with 2 sharp apical teeth and a deep excavation for apical maxillary palpomere at outer side. Maxillary palps 4-segmented, with apical palpomere distinctly widened, truncate apically in male; slightly widened in female. Antennae in male reaching elytral humeri; antennomere 1 robust; antennomere 2 shorter than 3; antennomere 3 longer than 4; antennomeres 7–11 each distinctly longer than wide; antennomere 11 longest, about 2.26 times as long as wide (Fig. 55). Antennae in female reaching pronotal base; antennomere 11 about 2.05 times as long as wide.

*Pronotum*. Lateral sides widest at or near base, roundly moderately narrowed anteriorly, anterior angles strongly produced (Fig. 10). Anterior and lateral margins bordered, lateral margins invisible in dorsal view. Trichobothria present on posterior angles. Disc covered with moderately dense punctures; lateral sides covered with much coarser punctures, becoming larger toward base, partially confluent near basal margin; interspaces covered with fine and sparse punctures. Scutellum slightly wider than long, narrowed posteriorly.

*Elytra*. Lateral sides moderately widened posteriorly, widest beyond middle, thence roundly narrowed posteriorly. Humeral calli well developed. Disc covered with 11 regular rows of large punctures, including a short scutellar row; sometimes punctures rather irregular between 6th and 8th striae in apical half; interspaces shagreened, covered with fine and sparse punctures. Epipleura wholly visible in lateral view. Hind wings well developed.

*Venter*. Hypomera weakly rugose, with a few punctures near anterolateral corners of prosternum. Prosternum covered with coarse and dense punctures bearing long setae; prosternal process enlarged apically, bordered laterally, with sparse punctures. Metasternum covered with small and sparse punctures in median region, large and dense punctures in lateral region. Abdominal ventrites covered with dense punctures bearing short setae.

*Legs*. Moderately robust. Tibiae widened apically, with a tooth-like projection. Fore legs with tarsomere 1 strongly enlarged, distinctly wider than 3 in male; very slightly narrower than 3 in female. Tarsal claws appendiculate.

*Genitalia*. Aedeagus rather thick, with short apical process in dorsal view; strongly curved, with apical process pointed and slightly bent downward at apex in lateral view (Figs 56–60). Spermatheca absent.

#### Distribution.

**Figure 62.**
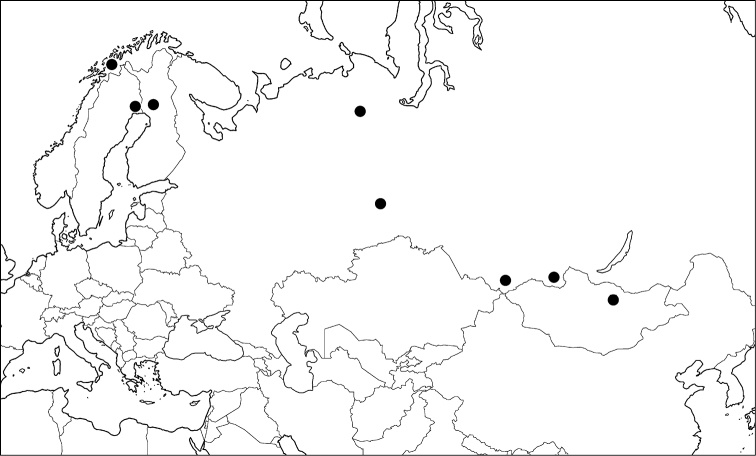
Distribution of *Gonioctena norvegica* based on specimens in the Palaearctic region.

Finland, Norway, Sweden, Russia (North European Territory, West Siberia), Mongolia (Fig. 62).

#### Host plant.

Salicaceae: *Salix* spp. (L. N. Medvedev and Voronova 1976, L. N. Medvedev and Zaytsev 1978, L. N. Medvedev and Roginskaya 1988).

#### Remarks.

The shape of aedeagus slightly varies geographically (Fig. 62). After examining the type and other specimens from the Palaearctic region, we conclude that *Gonioctena janovskii* from Mongolia should be synonymized with *Gonioctena norvegica*. Medvedev and Korotyaev (1980) synonymized *Gonioctena norvegica* with *Gonioctena affinis* (= *Gonioctena nivosa*), however *Gonioctena norvegica* differs in having shorter antennae and thicker aedeagus compared with those of *Gonioctena nivosa*, as previously mentioned by Silfverberg (1994b). Kippenberg (2010) treated *Gonioctena charitonowi* as a synonym of *Gonioctena norvegica*, however the illustration of the aedeagus of *Gonioctena charitonowi* looks quite different from that of *Gonioctena norvegica*. It should be re-examined to confirm its taxonomic status.

### Gonioctena (Gonioctena) springlovae

Taxon classificationAnimaliaColeopteraChrysomelidae

(Bechyně, 1948)

**Figures 63–65.**
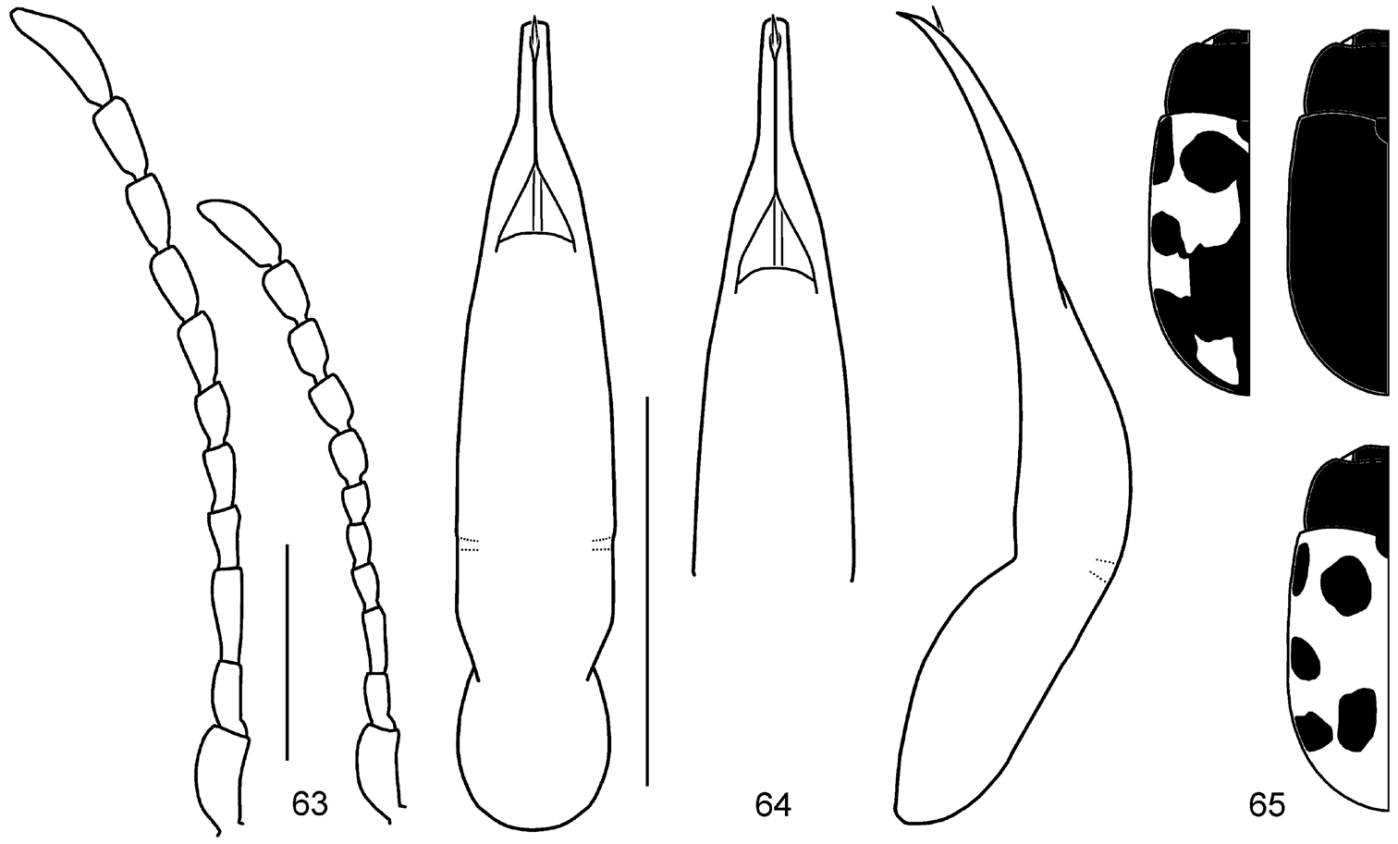
*Gonioctena springlovae*. **63** Antenna (♂, ♀) **64** Aedeagus **65** Color variation. Scale bars = 1.0 mm.

Figs 11–12 , 16 , 63–65

Phytodecta springlovae Bechyně, 1948: 115, 116 (type locality: Japonia, Kioto).
Gonioctena (Gonioctena) springlovae : Chûjô and Kimoto 1960: 5, 1961: 153; Gressitt and Kimoto 1963: 358, 362; Kimoto 1963: 17, 1964: 280, 282; Takizawa 1976: 454 (larva, pupa, biology); L. N. Medvedev 1992: 575 (as synonym of Gonioctena affinis); Kimoto and Takizawa 1994: 139, 229, 302, 452, 498 (incl. larva and pupa); V. L. Medvedev 2004: 41; Takizawa 2007: 38, 42; Kippenberg 2010: 434.
Gonioctena springlovae : Takizawa 1971: 173; Cox 1996: 146 (pupa); Kudo and Hasegawa 2003: 729 (biology); Takahashi 2012: 289.
Phytodecta gracilicornis ?: Jacoby 1885: 210 (misidentification).

#### Type material.

Holotype: ♂ (NMPC), Japon, Kioto // TYPUS // *Phytodecta* TYPE *springlovae* n. sp. 1945 Det. J. Bechyně. // Coll. Achard Mus. Pragense // Mus. Nat. Pragae Inv. 18 960 // Gonioctena (Gonioctena) springlovae (Bechyně) Det. S. GE 2004.

#### Other material.

**Japan (Hokkaido)**: 1♂ (NMPC), Japon, Kioto // TYPUS // Phytodecta springlovae ab. graduata n. ab. TYPE 1945 Det. J. Bechyně. // Gonioctena (Gonioctena) springlovae (Bechyně) Det. S. GE 2004; 1♂ (NMPC), Japon, Kioto // coll. Achard Mus. Pragense // TYPUS // Phytodecta springlovae ab. graduata n. ab. PARATYPE 1945 Det. J. Bechyně. // Mus. Nat. Pragae Inv. 18 961 // Gonioctena (Gonioctena) springlovae (Bechyně) Det. S. GE 2004; 3♂♂ (JBC), Japan, Hokkaido, Eniwa Mt. 30 km S from Sapporo, Shikotsu-Toya N.P. 500 m, 6.VII.1997, lgt. V. Kostal; 1♀ (BMNH), Japan, G. Lewis. 1910-320. // Chiuzenji; 5♂♂, 1♀ (HCC), Japan, Hokkaido, Sapporo, Kannon-zawa, 29.V.1995, S. Kudo; 1♂, 1♀ (HCC), Japan, Hokkaido, Sapporo, Jozankei, 29.VIII.2011, H. Suenaga leg.; 1♂ (SEHU), Jozankei, Hokkaido, 22.VII.1955, M. Konishi; 1♂, 1♀ (SEHU), Hidaka, Hokkaido, 1955, S. Watanabe; 1♂ (AWC), JAPAN, Takahiro Parking Area, Bifuka, Hokkaido, 4.VII.2002, Y. Komiya lgt. **Russia (Sakhalin)**: 1♂ (NHMB), Sakhalin, riv. Naiba, VIII.1991; 2♂♂ (BMNH), Russia, Saghalien, Central Expt. Sta.; 1♂ (SEHU), Saghalien, 16.VII.1933, Uchida, Okada, Sawamoto & Hoye legs; 4♂♂ (ZIN), Russia, Sakhalin Oblast, Sakhalin Island, Holmsky pass (approx. 47°N, 142°E), 1.VII.1982, Smirnov leg.

#### Diagnosis.

See diagnosis of *Gonioctena gracilicornis*.

#### Redescription.

Measurements in mm (n = 5): length of body: 5.70–6.20 (mean 6.00); width of body: 3.20–3.60 (mean 3.40); height of body: 2.30–2.50 (mean 2.42); width of head: 1.60–1.75 (mean 1.66); interocular distance: 1.10–1.20 (mean 1.13); width of apex of pronotum: 1.87–2.05 (mean 1.95); width of base of pronotum: 2.70–3.05 (mean 2.84); maximum width of pronotum: 2.72–3.07 (mean 2.87); length of pronotum along midline: 1.35–1.50 (mean 1.41); length of elytra along suture: 4.10–4.60 (mean 4.42).

Body oblong oval and moderately convex (Fig. 11). Coloration variable. Head black, with dark reddish brown band near apex of mandibles. Antennomeres 1–5 yellowish brown, generally darkened, 6–7 dark brown to blackish brown, 8–11 black. Pronotum entirely black. Scutellum black. Elytra reddish brown or yellowish brown, with 5 pairs of black spots, generally connected with each other, rarely elytra entirely black (Fig. 65). Venter black, with lateral margins of last abdominal ventrite reddish brown. Legs black, with tarsi blackish brown, sometimes tibiae largely dark brown to reddish brown.

**Figures 66–67.**
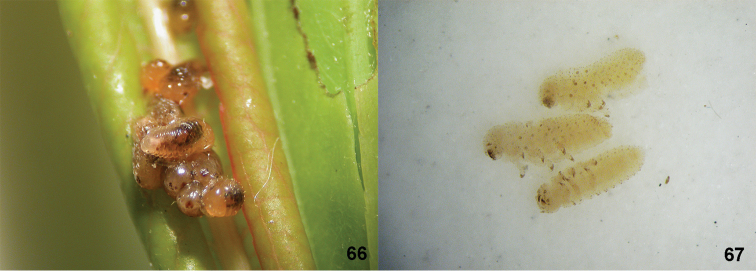
Ovoviviparous species. **66** Newly laid larvae of *Gonioctena gracilicornis* **67** Larvae dissected from a female of *Gonioctena nivosa*.

*Head*. Vertex weakly convex, covered with sparse punctures, becoming coarser and denser toward sides. Frontal suture V-shaped, coronal suture absent. Frons flat, strongly depressed anteriorly, covered with moderately dense punctures. Clypeus narrow and trapezoidal. Anterior margin of labrum distinctly concave. Mandibles with 2 sharp apical teeth and a deep excavation for apical maxillary palpomere at outer side. Maxillary palps 4-segmented, with apical palpomere distinctly widened, truncate apically in male; slightly widened in female. Antennae in male longer than half length of body; antennomere 1 robust; antennomere 2 shorter than 3; antennomere 3 longer than 4; antennomeres 7–11 elongate; antennomere 11 longest, about 3.95 times as long as wide (Fig. 63). Antennae in female reaching elytral humeri; antennomere 11 about 2.58 times as long as wide.

*Pronotum*. Lateral sides widest near base, feebly rounded, slightly narrowed anteriorly, anterior angles strongly produced (Fig. 12). Anterior and lateral margins bordered, lateral margins well visible in dorsal view. Trichobothria present on posterior angles. Disc covered with sparse punctures; lateral sides covered with much coarser and denser punctures, becoming larger toward base, partially confluent near basal margin; interspaces covered with fine and sparse punctures. Scutellum variable in length, as long as wide, longer than wide or wider than long.

*Elytra*. Lateral sides slightly widened posteriorly, widest beyond middle, thence roundly narrowed posteriorly. Humeral calli well developed. Disc covered with 11 regular rows of large punctures, including a short scutellar row; interspaces shagreened, covered with fine and sparse punctures. Epipleura wholly visible in lateral view. Hind wings well developed.

*Venter*. Hypomera weakly rugose, with dense punctures on anterior side. Prosternum covered with coarse and dense punctures bearing long setae; prosternal process enlarged apically, bordered laterally, with sparse punctures. Metasternum covered with small and sparse punctures in median region, large and dense punctures in lateral region. Abdominal ventrites covered with dense punctures bearing short setae.

*Legs*. Moderately robust. Tibiae widened apically, with a tooth-like projection. Fore legs with tarsomere 1 enlarged, slightly wider than 3 in male; distinctly narrower than 3 in female. Tarsal claws appendiculate.

*Genitalia*. Aedeagus thin, distinctly narrowed apically, with apical process thin in dorsal view; moderately curved, with apical process pointed and slightly bent downward at apex in lateral view (Fig. 64). Spermatheca absent.

#### Distribution.

Russia (Sakhalin), Japan (Hokkaido) (Fig. 16).

#### Host plant.

Salicaceae: *Populus* spp., *Salix* spp. (Chûjô and Kimoto 1961, Kimoto 1964); *Salix* spp. (Takizawa 1976, Kudo and Hasegawa 2003); *Populus maximowiczii*, *Salix* spp. (Takizawa 2007).

#### Remarks.

*Gonioctena springlovae* is restricted to Hokkaido and Sakhalin, whereas its closely related species *Gonioctena gracilicornis* is widely distributed in the Northeastern Palearctic region. The distributions of these two species overlap only in southern Sakhalin (Fig. 16). The type locality “Kioto [= Kyoto in Honshu]” is probably in error. As Chûjô and Kimoto (1960) mentioned, no single specimen has been collected again in Honshu whereas many specimens have been collected in Hokkaido. This species is ovoviviparous (Takizawa 1976, Kimoto and Takizawa 1994, Kudo and Hasegawa 2003).

## Supplementary Material

XML Treatment for Gonioctena (Gonioctena) amurensis

XML Treatment for Gonioctena (Gonioctena) gracilicornis

XML Treatment for Gonioctena (Gonioctena) jani

XML Treatment for Gonioctena (Gonioctena) nivosa

XML Treatment for Gonioctena (Gonioctena) norvegica

XML Treatment for Gonioctena (Gonioctena) springlovae

## References

[B1] Achard J (1924) Les *Phytodecta* et leurs variations. Espèces et variétés nouvelles. Rectifications synonymiques. Časopis Československé Společnosti Entomologické 21: 31–37.

[B2] Baly JS (1862) Descriptions of new species of phytophagous beetles. The Annals and Magazine of Natural History 10(3): 17–29.

[B3] Bechyně J (1948) Příspěvek k poznání rodu *Phytodecta* Kirby. Additamenta ad cognitionem specierum generis *Phytodecta* Kirby (Col. Phytoph. Chrysomelidae). Sborník Národního Musea v Praze 3B(3) [1947]: 89–158, 5 pls.

[B4] Bieńkowski AO (2004) Leaf-beetles (Coleoptera: Chrysomelidae) of Eastern Europe. New key to subfamilies, genera, and species. Mikon-print, Moscow, 278 pp.

[B5] Brown WJ (1942) The American species of *Phytodecta* Kby. (Coleoptera, Chrysomelidae). The Canadian Entomologist 74: 99–105. doi: 10.4039/ent7499-6

[B6] Brown WJ (1952) Some species of Phytophaga (Coleoptera). The Canadian Entomologist 84: 335–342. doi: 10.4039/ent84335-11

[B7] Cantonnet F (1968) Révision des espèces françaises du genre *Phytodecta* et description d’une espèce nouvelle (Col. Chrysomelidae). L’Entomologiste 24: 38–49.

[B8] Chen SH (1935) Classfication of Asiatic *Phytodecta* (Col. Chrysomelinae). The Chinese Journal of Zoology 1: 125–133.

[B9] Chen SH (1936) Catalogue des Chrysomelinae de la Chine, de l’Indochine et du Japon. Notes d’Entomologie Chinoise 3: 63–102.

[B10] Chevrolat LAA (1836) Chrysomelidae [Livraison 5]. In: Dejean PFMA (Ed.) Catalogue des coléoptères de la collection de le M. le Comte Dejean. Deuxième édition, revue, corrigée et augmentée Méquignon-Marvis Père et Fils, Paris, 361–443.

[B11] Cho HW (2016) Revision and classification of the genus *Gonioctena* Chevrolat, 1836 (Coleoptera: Chrysomelidae: Chrysomelinae). PhD thesis, University of Wrocław, Wrocław, Poland.

[B12] Cho HW Borowiec L (2016) On the genus *Gonioctena* Chevrolat (Coleoptera: Chrysomelidae: Chrysomelinae), with descriptions of seven new species from the Oriental region and Palaearctic China. Zootaxa 4067: 168–184. doi: 10.11646/zootaxa.4067.2.3 10.11646/zootaxa.4067.2.3 27395869

[B13] Cho HW Lee JE (2008) Taxonomic review of the genus *Gonioctena* Chevrolat (Coleoptera: Chrysomelidae: Chrysomelinae) in the Korean Peninsula. Entomological Research 38: 104–113. doi: 10.1111/j.1748-5967.2008.00145.x

[B14] Cho HW Lee JE (2010) *Gonioctena koryeoensis* (Coleoptera: Chrysomelidae: Chrysomelinae), a new species from Korea, with a description of immature stages. Zootaxa 2438: 52–60. doi: 10.11646/%25x

[B15] Chûjô M (1941) Chrysomelid-beetles from Korea (III). Transactions of the Natural History Society of Formosa 31: 61–75.

[B16] Chûjô M Kimoto S (1960) Descriptions of three new genera and a new species of chrysomelid-beetles from Japan, with some notes on the Japanese species. Niponius, Acta Coleopterologica 1(4): 1–10.

[B17] Chûjô M Kimoto S (1961) Systematic catalog of Japanese Chrysomelidae (Coleoptera). Pacific Insects 3: 117–202.

[B18] Clark SM LeDoux DG Seeno TN Riley EG Gilbert AJ Sullivan JM (2004) Host plants of leaf beetle species occurring in the United States and Canada. The Coleopterists Society, Special Publication No. 2, 476 pp.

[B19] Cox ML (1996) The Pupae of Chrysomeloidea. In: Jolivet PHA Cox ML (Eds) Chrysomelidae Biology. Vol. 1: The Classification, Phylogeny and Genetics. SPB Academic Publishing, Amsterdam, 119–265.

[B20] Crotch GR (1873) Materials for the study of the Phytophaga of the United States. Proceedings of the Academy of Natural Sciences of Philadelphia 25: 19–83.

[B21] Daccordi M Lavarini N Ruffo S (1991) Considerazioni faunistiche e biogeografiche sulle *Gonioctena* italiane (Coleoptera: Chrysomelidae). Atti XVI Congresso Nazionale Italiano di Entomologia. Bari - Martina Franca (Ta): 93–101.

[B22] Dubeshko LN Medvedev LN (1989) Ecology of Chrysomelidae from Siberia and Far East. Irkutsk University press, Irkutsk, 224 pp [In Russian]

[B23] Dufour LJM (1851) Des zones entomologique dans nos Pyrénées occidentales, et designation des insectes qui les habitent (2). Actes de la Société Linnéenne de Bordeaux 17: 304–364.

[B24] Fabricius JC (1787) Mantissa insectorum, sistens eorum species nuper deietes adiectis characteribus genericis, differentiis specificis, emendationibus, observationibus. Tom. I Christ. Gottl. Proft, Hafniae, xx + 348 pp. doi: 10.5962/bhl.title.36471

[B25] Gradl H (1882) Aus der Fauna des Egerlands. Zweite Folge. Entomologische Nachrichten 8: 323–332.

[B26] Gressitt JL Kimoto S (1963) The Chrysomelidae (Coleopt.) of China and Korea, Part 2. Pacific Insects Monograph 1B: 301–1026.

[B27] Gyllenhal L (1808) In: Schoenherr CJ (Ed.) Synonymia Insectorum, oder: Versuch einer Synonymie aller bisher bekannten lnsecten; nach Fabricii Systema Eleutheratorum & c. geordnet. Erster Band. Eleutherata oder Käfer. Zweiter Theil. Sprercheus–Cryptocephalus. C. F. Marquard, Stockholm, ix + 424 pp., 1 pl.

[B28] Heyden L von (1883) *Phytodecta affinis* und Verwandte. Entomologische Nachrichten 9: 52–53.

[B29] Holdhaus K Lindroth CH (1939) Die europäischen Koleopteren mit boreoalpiner Verbreitung. Annalen des Naturhistorischen Museums in Wien 50: 123–293.

[B30] International Commission on Zoological Nomenclature (1999) International code of zoological nomenclature. Fourth Edition The International Trust for Zoological Nomenclature, London. 10.3897/zookeys.931.51583 PMC7205856 32405237

[B31] Jacobson G (1901) Symbola ad cognitionem Chrysomelidarum Rossicae asiaticae. Öfversigt af Finska Vetenkaps-Societetens Förhandlingar 43[1900–1901]: 99–147.

[B32] Jacoby M (1885) Descriptions of the phytophagous Coleoptera of Japan, obtained by Mr. George Lewis during his Second Journey, from February 1880 to September 1881. Part I. Proceedings of the Scientific Meetings of the Zoological Society of London 1885: 190–211. doi: 10.1111/j.1096-3642.1885.tb02894.x

[B33] Jolivet P (1973) Essai d’analyse écologique de la faune Chrysomélidienne de la Corée. Cahiers du Pacifique 17: 253–288.

[B34] Kimoto S (1963) Descriptions of new species of the Chrysomelidae from Japan, with notes on some known species. Fragmenta Coleopterologica 3/4: 13–18.

[B35] Kimoto S (1964) The Chrysomelidae of Japan and the Ryukyu Islands V. Subfamily Chrysomelinae. Journal of the Faculty of Agriculture, Kyushu University 13: 263–286.

[B36] Kimoto S Kawase E (1966) A list of some chrysomelid specimens collected in E. Manchuria and N. Korea. Esakia 5: 39–48.

[B37] Kimoto S Takizawa H (1994) Leaf beetles (Chrysomelidae) of Japan. Tokai University Press, Tokyo, 539 pp.

[B38] Kippenberg H (1994) Familie: Chrysomelidae. In: Lohse GA Lucht WH (Eds) Die Käfer Mitteleuropas. Band 14. Supplementband mit Katalogteil. Goecke & Evers, Krefeld, 17–92, 142.

[B39] Kippenberg H (2010) New acts and comments, Chrysomelidae: Chrysomelinae. In: Löbl I Smetana A (Eds) Catalogue of Palaearctic Coleoptera, Volume 6, Chrysomeloidea. Apollo Books, Stenstrup, 67–73, 390–443.

[B40] Kittel G (1884) Systematische Uebersicht der Käfer, welche in Baiern und der nächsten Umgebung vorkommen. (Fortsetzung). Correspondenz-Blatt des naturwissenschaftlichen Vereines in Regensburg 38: 18–32.

[B41] Kraatz G (1879a) Über die Verwandten der *Phytodecta viminalis* L. Zeitschrift für Entomologie (N. F.) 7: 45–56.

[B42] Kraatz G (1879b) Neue Käfer vom Amur. Deutsche Entomologische Zeitschrift 23: 121–144, pl. II. doi: 10.1002/mmnd.48018790117

[B43] Kudo S Hasegawa E (2003) Diversified reproductive strategies in *Gonioctena* (Chrysomelinae) leaf beetles. In: Jolivet P Santiago-Blay JA Schmitt M (Eds) New Developments in the Biology of Chrysomelidae, SPB Academic Publishing, The Hague, 727–738.

[B44] Lee JE An SL (2001) Family Chrysomelidae. Economic Insects of Korea 14, Insecta Koreana Supplement 21. National Institute of Agricultural Science and Technology, Suwon, 231 pp [In Korean]

[B45] Letzner K (1864) Bericht über die Thätigkeit der entomologischen Section der Schlesischen Gesellschaft im Jahre. Jahresbericht der Schlesischen Gesellschaft für vaterländische Kultur 42: 141–146.

[B46] Li JK (1992) The Coleoptera fauna of northeast China. Education Publishing House, Jilin, 205 pp [In Chinese]

[B47] Lopatin IK Aleksandrovich OR Konstantinov AS (2004) Check list of leaf-beetles (Coleotera, Chrysomelidae) of the Eastern Europe and Northern Asia. Mantis Publishing House, Olsztyn, 343 pp.

[B48] Mannerheim CG (1852) Zweiter Nachtrag zur Kaefer-Fauna der nord-amerikanischen Laender des Russischen Reiches. Bulletin de la Société Impériale des Naturalistes de Moscou 25(2): 283–387.

[B49] Mannerheim CG (1853) Dritter Nachtrag zur Kaefer-fauna der Nord-Amerikanischen Laender des Russischen Reiches. Bulletin de la Société Impériale des Naturalistes de Moscou 26(3): 95–273.

[B50] Marseul S (1888) Monographie des chrysomélides de l’Ancien-Monde (Suite). L’Abeille, Journal d’Entomologie 25 [1887–1888]: 1–96 [299–394].

[B51] Medvedev LN (1963) On the fauna of Chrysomelidae (Coleoptera) from Kamchatka. In: Fauna of Kamchatka. Izdatelstvo Akademii nauk SSSR, Moskva-Leningrad, 113–117. [In Russian]

[B52] Medvedev LN (1968) In: Ivliev LA Kononov DG Medvedev LN, Fauna of leaf beetles of Magadan Province and northern Khabarovsk Territory. In: Kurentsov AI Konovalova ZA (Eds) Fauna and ecology of the insects of Far East. Akademiya Nauk SSSR, Sibirskoe otdelenie, Vladivostok, 62–87. [In Russian]

[B53] Medvedev LN (1976) In: Medvedev LN Voronova NV On the fauna of chrysomelid beetles (Coleoptera, Chrysomelidae) of Mongolia. Nasekomye Mongolii 4: 222–236. [In Russian]

[B54] Medvedev LN (1982) The leaf beetles of the Mongolian People’s Republic, Identification book, Moscow, 303 pp [In Russian]

[B55] Medvedev LN (1992) Family Chrysomelidae. In: Ler PA (Ed.) Opredelitel’ Nasekomykh Dal’nego Vostoka SSSR Tom 3 - Zheskokrylye, ili zhuki Chast 2. Nauka, St. Petersburg, 533–602. [In Russian]

[B56] Medvedev LN (2006a) Contribution to the fauna of leaf-beetles (Coleoptera, Chrysomelidae) of Amur Oblast’. Euroasian Entomological Journal 5: 137–143. [In Russian]

[B57] Medvedev LN (2006b) To the Knowledge of Chrysomelidae (Coleoptera) described by V. Motschulsky. Russian Entomological Journal 15: 409–417.

[B58] Medvedev LN (2014) Fauna of leaf beetles (Insecta: Coleoptera: Chrysomelidae) from Siberia and Far East of Russia. Regional Problems 17: 35–39. [In Russian]

[B59] Medvedev LN Dubeshko LN (1992) Identification key of leaf-beetles of Siberia. Izdatelstvo Irkutskogo Universiteta, Irkutsk, 220 pp [In Russian]

[B60] Medvedev LN Korotyaev B (1980) Notes on leaf beetles (Coleoptera, Chrysomelidae) of Arctic Asia and Kamchatka. In: Entomological Investigations of North-East USSR. Far East Scientific Center, Vladivostok, 77–95. [In Russian]

[B61] Medvedev LN Roginskaya EY (1988) Catalogue of the host plants of USSR leaf beetles. Academy of Sciences of the USSR, Institute of Evolutionary Morphology and Ecology of Animals, Moscow, 192 pp [In Russian]

[B62] Medvedev LN Voronova NV (1976) On the fauna of chrysomelid beetles (Coleoptera, Chrysomelidae) of Mongolia. Nasekomye Mongolii 4: 222–236. [In Russian]

[B63] Medvedev LN Zaytsev YM (1978) Larvae of leaf beetles of Siberia and the Far East. Nauka, Moscow, 182 pp [In Russian]

[B64] Medvedev LN Zaytsev (1980) New data on chrysomelid-beetle larvae from Mongolia (Coleoptera, Chrysomelidae). Insects of Mongolia 7: 97–106. [In Russian]

[B65] Medvedev VL (1999) New Oriental forms of the genus *Gonioctena* Chevrolat (Coleoptera: Chrysomelidae: Chrysomelinae). Serangga 4: 11–16.

[B66] Medvedev VL (2003) Variability of the integument pattern in leaf-beetles for the example of species of the genus *Gonioctena* Chevrolat (Coleoptera, Chrysomelidae). Entomological Review 83: 639–647.

[B67] Medvedev VL (2004) New species of the genus *Gonioctena* Chevrolat, 1837 (Coleoptera: Chrysomelidae) from China. Russian Entomological Journal 13: 41–42.

[B68] Mikhailov YE (2001) Significance of colour polymorphism in mountain populations of abundant leaf beetles (Coleoptera, Chrysomelidae). Pirineos 156: 57–68. doi: 10.3989/pirineos.2001.v156.80

[B69] Mikhailov YE Hayashi M (2000) Chrysomelidae of Sakhalin I. Entomological Review of Japan 55: 71–83.

[B70] Mohr KH (1966) 88. Familie: Chrysomelidae. In: Freude H Harde KW Lohse GA (Eds) Die Käfer Mitteleuropas. Band 9. Cerambycidae, Chrysomelidae. Goecke & Evers, Krefeld, 95–280.

[B71] Motschulsky V (1860) Coléoptères rapportés de la Siberie orientale et notamment des pays situées sur les bors du fleuve Amour par MM Schrenck, Maack, Ditmar, Voznessenski etc. In: Schrenck L von (Ed.) Reisen und Forschungen im Amur-Lande in den Jahren 1854–1856. Band II. Zweite Lieferung, St. Petersburg, 77–258, pls VI–XI.

[B72] Notman H (1921) Concerning species, with notes on *Phytodecta affinis* Gyll. and *pallidus* Linn. Bulletin of the Brooklyn Entomological Society 16: 75–78.

[B73] Palmén E (1946) Zur Systematik finnischer Chrysomeliden. 3. Untergattung *Phytodecta* s. str. Annales Entomologici Fennici 11: 227–234.

[B74] Pic M (1924) Notes diverses, descriptions et diagnoses. L’Échange, Revue Linnéenne 39 [= 40]: 18–19, 21–23, 25–27, 29–30.

[B75] Reitter E (1913) Fauna Germanica. Die Käfer des Deutschen Reiches. Nach der analytischen Methode bearbeitet. IV. Band. [1912]. K. G. Lutz’ Verlag, Stuttgart, 236 pp., pls. 129–152.

[B76] Riley EG Clark SM Seeno TN (2003) Catalog of the leaf beetles of America north of Mexico (Coleoptera: Megalopodidae, Orsodacnidae and Chrysomelidae, excluding Bruchinae). Coleopterists Society, Special Publication no. 1, 290 pp.

[B77] Ronchetti V (1922) Al Monte Cevedale (m 3774) per la parete Sud-Ovest. Rivista Mensile del Club Alpino Italiano 41: 86–89.

[B78] Sahlberg J (1887) Coleoptera och Hemiptera, insamlade af Vega-expeditionens medlemmar a Berings sunds amerikanska kust uti omgifningarna af Port Clarence, vid Grantley Harbour och sjon Iman-Ruk den 23–26 juli 1879. In: Nordenskiold AE (Ed.) Vega-Expeditionens Vetenskapliga Iakttagelser bearbetade af deltagare i resan och andra forskare. Fjerde Bandet. F. G. Beijers Forlag, Stockholm, 43–57.

[B79] Schaeffer C (1924) On a few new and old Chrysomelidae. Journal of the New York Entomological Society 32: 138–145.

[B80] Silfverberg H (1977) Nomenclatoric notes on Coleoptera Polyphaga. Notulae Entomologicae 57: 91–94.

[B81] Silfverberg H (1992) Enumeratio Coleopterorum Fennoscandiae, Daniae et Baltiae. Helsingin Hyönteisvaihtoyhdistys, Helsinki, 94 pp.

[B82] Silfverberg H (1994a) Chrysomelidae in the Arctic. In: Jolivet PH Cox ML Petitpierre E (Eds) Novel Aspects of the Biology of Chrysomelidae. Kluwer Academic Publishers, Dordrecht, 503–510. doi: 10.1007/978-94-011-1781-4_37

[B83] Silfverberg H (1994b) Colour variation in Finnish Chrysomelidae (Coleoptera). 2. The genus *Gonioctena*. In: Furth DG (Ed.) Proceedings of the third International Symposium on the Chrysomelidae, Beijing 1992. Backhuys, Leiden, 31–37.

[B84] Silfverberg H (2004) Enumeratio nova Coleopterorum Fennoscandiae, Daniae et Baltiae. Sahlbergia 9: 1–111.

[B85] Stål C (1865) Monographie des Chrysomélides de l’Amérique. III. Nova Acta Societatis Scientiarum upsaliensis (3) 4: 177–365.

[B86] Steinhausen WR (1994) Familie Chrysomelidae. In: Klausnitzer B (Ed.) Die Larven der Käfer Mitteleuropas, 2 Band Goecke & Evers, Krefeld, 231–314.

[B87] Steinhausen WR (1996) Status of west Palaearctic leaf beetle larvae research. In: Jolivet PHA Cox ML (Eds) Chrysomelidae Biology. Vol. 3: General Studies. SPB Academic Publishing, Amsterdam, 65–91.

[B88] Strand A (1936) *Phytodecta norvegicus* n. sp. (Col., Chrysomelidae). Norsk Entomologisk Tidsskrift 4: 104–105.

[B89] Suffrian E (1851) Zur Kenntniss der Europäischen Chrysomelen. Linnaea Entomologica 5: 1–280.

[B90] Suffrian E (1858) Uebersicht der in den Verein. Staaten von Nord-Amerika einheimischen Chrysomelen. Entomologische Zeitung (Stettin) 19: 381–400.

[B91] Székessy W (1934) Ein neuer Fall von boreoalpiner Verbreitung bei Koleopteren. *Phytodecta nivosa* Suffr. = *Phytodecta affinis* Gyllh. (Chrysom.). Nebst einem Nachtrag zu Holdhaus’ Verzeichnis der boreoalpinen Tierformen. Koleopterologische Rundschau 20: 32–36.

[B92] Takahashi S (2012) Chrysomelidae (exclusive of Bruchinae & Donaciinae). In: Shiyake S (Ed.) Specimen list of Coleoptera in the Insect Collection of the Osaka Museum of Natural History (Part 2). Osaka Museum of Natural History, Osaka, 239–372.

[B93] Takizawa H (1971) A list of chrysomelid beetles from Sakhalin in the collection of the Entomological Institute, Hokkaido University (Coleoptera). Kontyû 39: 172–176.

[B94] Takizawa H (1976) Larvae of the genus *Gonioctena* Chevrolat (Coleoptera, Chrysomelidae): descriptions of Japanese species and the implications of larval characters for the phylogeny. Kontyû 44: 444–468.

[B95] Takizawa H (1985) Notes on Korean Chrysomelidae, part 2. Nature and Life (Kyungpook Journal Biological Sciences) 15(1): 1–18.

[B96] Takizawa H (2007) A revision of the genus *Gonioctena* Chevrolat in Japan (Coleoptera: Chrysomelidae). Insecta Matsumurana, new series 63: 35–50.

[B97] Warchałowski A (1994) Chrysomelidae. Stonkowate (Insecta: Coleoptera). Część IV (podplemiona: Chrysomelina, Gonioctenina, Phratorina i Entomoscelina oraz podrodzina Galerucinae. Fauna Polski, tom 16. Muzeum i Instytut ZoologiiPAN, Warszawa, 302 pp [In Polish]

[B98] Warchałowski A (2003) Chrysomelidae. The leaf-beetles of Europe and the Mediterranean area. Natura Optima Dux Foundation, Warszawa, 600 pp.

[B99] Warchałowski A (2010) The Palaearctic Chrysomelidae. Identification keys. Volume 1. Natura Optima Dux Foundation, Warszawa, 629 pp.

[B100] Weise J (1884) Chrysomelidae. Lieferung 3. In: Naturgeschichte der Insekten Deutschlands. Erste Abteilung Coleoptera. Sechster Band. Nicolaische Verlags-Buchhandlung, Berlin, 369–568.

[B101] Weise J (1891) Ueber Varietäten von *Phytodecta*. Deutsche Entomologische Zeitschrift 35: 160.

[B102] Weise J (1893) Chrysomelidae. Lieferung 6. In: Naturgeschichte der Insecten Deutschlands. Erste Abtheilung Coleoptera. Sechster Band. Nicolaische Verlags-Buchhandlung, Berlin, 961–1161.

[B103] Weise J (1906) Chrysomelidae. In: Heyden L von Reitter E Weise J (Eds) Catalogus Coleopterorum Europae, Caucasi et Armeniae Rossicae. Friedländer & Sohn, Berlin, Edmund Reitter, Paskau, Revue d’Entomologie, Caen, 533–585.

[B104] Weise J (1916) Pars 68: Chrysomelidae: 12. Chrysomelinae. In: Schenkling S (Ed.) Coleopterorum Catalogus. W. Junk, Berlin, 255 pp.

[B105] Wilcox JA (1972) A review of the North American Chrysomeline leaf beetles (Coleoptera: Chrysomelidae). New York State Museum Science Service Bulletin 421: 1–37 + 162 figs.

[B106] Winkelman J Debreuil M (2008) Les Chrysomelinae de France (Coleoptera, Chrysomelidae). Supplément Rutilans, 188 pp.

[B107] Winkler A (1930) Catalogus Coleopterorum regionis palaearcticae. Pars 11. Albert Winkler, Wien, 1265–1392.

[B108] Yang XK Ge SQ Wang SY Li WZ Cui JZ (2014) Fauna Sinica, Insecta Vol. 61. ColeopteraChrysomelidaeChrysomelinae. Science Press, Beijing, 641 pp [In Chinese]

[B109] Yang XK Ge SQ Nie RE Ruan YG Li WZ (2015) Chinese leaf beetles. Science Press, Beijing, 507 pp. + 83 plates.

[B110] Zaytsev YM Medvedev LN (1977) Some chrysomelid-beetle larvae from Mongolia (Coleoptera, Chrysomelidae). In: Insects of Mongolia. Nauka, Leningrad, 5: 353–371. [In Russian]

[B111] Zaytsev YM Medvedev LN (2009) Leaf beetle larvae of Russia. KMK Scientific Press, Moscow, 246 pp. [In Russian]

